# NADPH Oxidases Are Required for Full Platelet Activation In Vitro and Thrombosis In Vivo but Dispensable for Plasma Coagulation and Hemostasis

**DOI:** 10.1161/ATVBAHA.120.315565

**Published:** 2020-12-03

**Authors:** Dina Vara, Reiner K. Mailer, Anuradha Tarafdar, Nina Wolska, Marco Heestermans, Sandra Konrath, Manuela Spaeth, Thomas Renné, Katrin Schröder, Giordano Pula

**Affiliations:** 1 Institute of Biomedical and Clinical Science, University of Exeter Medical School, United Kingdom (D.V.).; 2 Institute of Clinical Chemistry and Laboratory Medicine, University Medical Center Hamburg-Eppendorf, Germany (R.K.M., N.W., M.H., S.K., T.R., G.P.).; 3 Cancer Research UK Manchester Institute, University of Manchester (A.T.).; 4 Institute of Cardiovascular Physiology, Goethe-University, Frankfurt, Germany (M.S., K.S.).

**Keywords:** blood platelets, chlorides, NADPH oxidases, oxidation-reduction, thrombosis

## Abstract

Using 3KO (triple NOX [NADPH oxidase] knockout) mice (ie, NOX1^−/−^/NOX2^−/−^/NOX4^−/−^), we aimed to clarify the role of this family of enzymes in the regulation of platelets in vitro and hemostasis in vivo.

**Approach and Results:**

3KO mice displayed significantly reduced platelet superoxide radical generation, which was associated with impaired platelet aggregation, adhesion, and thrombus formation in response to the key agonists collagen and thrombin. A comparison with single-gene knockouts suggested that the phenotype of 3KO platelets is the combination of the effects of the genetic deletion of NOX1 and NOX2, while NOX4 does not show any significant function in platelet regulation. 3KO platelets displayed significantly higher levels of cGMP—a negative platelet regulator that activates PKG (protein kinase G). The inhibition of PKG substantially but only partially rescued the defective phenotype of 3KO platelets, which are responsive to both collagen and thrombin in the presence of the PKG inhibitors KT5823 or Rp-8-pCPT-cGMPs, but not in the presence of the NOS (NO synthase) inhibitor L-NG-monomethyl arginine. In vivo, triple NOX deficiency protected against ferric chloride–driven carotid artery thrombosis and experimental pulmonary embolism, while hemostasis tested in a tail-tip transection assay was not affected. Procoagulatory activity of platelets (ie, phosphatidylserine surface exposure) and the coagulation cascade in platelet-free plasma were normal.

**Conclusions:**

This study indicates that inhibiting NOXs has strong antithrombotic effects partially caused by increased intracellular cGMP but spares hemostasis. NOXs are, therefore, pharmacotherapeutic targets to develop new antithrombotic drugs without bleeding side effects.

## Abstract 2

Supplemental Digital Content is available in the text.

**Highlights**

- Triple NOX (NADPH oxidase) mice are defective in both collagen and thrombin responses.
- NOX4 has a negligible role in platelet regulation.
- cGMP increase and PKG (protein kinase G) activation are partially responsible for the defective phenotypes of 3KO (triple NOX knockout) platelets.
- Despite the refractoriness of 3KO platelets, coagulatory responses are not affected and the mice do not show increased bleeding.
- Overall, our data suggest that pharmacological inhibition of NOXs can become an effective and safe antiplatelet strategy.

The importance of NOX (NADPH oxidase)-dependent reactive oxygen species for platelet signaling and function has been investigated in an array of recent studies.^1–7^ In addition to their role in healthy platelet signaling, there is evidence that NOX activity sensitizes platelets and induces a state of hyperresponsiveness typical for thrombotic diseases.^5,8–11^ Although the activation mechanisms and the precise roles of NOXs in response to different platelet agonists and physiopathological conditions remain to be fully elucidated, NOXs are promising antiplatelet drug targets.^12–16^

Nonetheless, current literature remains inconclusive regarding several aspects of the function of NOXs in platelets. There is considerable disagreement on the specific roles of individual NOX family members in platelet signaling cascades. Although previous studies described a central role for NOX2 in the activation of platelets by collagen or collagen-related peptide,^6,17,18^ recent data suggest a limited role of this enzyme in the activation of platelets and the promotion of arterial thrombosis in vivo.^19^ In accordance with previous studies,^7,12^ we found NOX2 activity to be required for full platelet activation by thrombin but dispensable for collagen-induced responses.^5^ In parallel, we identified NOX1 to be essential for collagen signaling.^5,7^ Also for NOX1, there are discrepancies in literature. In parallel to studies showing a selective role for this enzyme in the signaling of collagen,^7,11,12^ NOX1 has also been described as a critical mediator for G-protein–dependent signaling of thrombin or thromboxane A2.^6^ Finally, in addition to NOX1^12^ and NOX2,^20^ a recent study reported NOX4 expression in human and mouse platelets,^6^ which may complicate the interpretation of existing data. To our knowledge, there are no platelet studies on *NOX4*^−/−^ mice or using selective NOX4 inhibitors.

A potential weakness in our understanding of the role of NOXs in hemostasis and thrombosis is that all existing studies are based on single gene-deficient models (*Nox1*^−/−^^5,6^ or *Nox2*^−/−^^5–8,19^). Due to overlapping biochemical functions of different NOXs, genetic knockout of a single NOX can be compensated by coexpressed NOX isoenzymes. This is a common phenomenon in genetically modified experimental models.^21^ In addition, single-gene knockout models have the important limitation of not being predictive of the effect on hemostasis and thrombosis of current NOX inhibitors, which are characterized by poor or lack of selectivity (ie, they inhibit multiple NOX family members simultaneously).^22,23^ Existing nonselective NOX inhibitors have so far been evaluated on platelets only ex vivo.^7,11,12,24^

To address the limitations of our understanding of the role of NOXs in platelet and hemostasis regulation, here we have characterized platelets and coagulation responses of mice deficient in NOX1, NOX2, and NOX4 ex vivo. The transgenic animals used in this study were generated by combined genetic deletion of *Nox1*, *Nox2* (*Cybb*), and *Nox4* and displayed no vascular abnormality or health problem in previous studies.^25^ Taken together, this study provides novel mechanistic information on the regulation of platelets by NOXs and the role of these enzymes in the regulation of hemostasis and thrombosis and in vivo. This information is essential for the development of NOX inhibitors for cardiovascular use.

## Methods

The data that support this study are available from the corresponding author upon request.

### Animals, Blood Collection, and Washed Platelet Preparation

3KO (triple NOX knockout) mice were generated by crossbreeding *Nox1*^−/−^,^26^ *Nox2 (Cybb*)^−/−^,^27^ and *Nox4*^−/−^^28^ mice and were backcrossed for at least 10 generations into the C57BL/6J background, as described previously.^25^ Considering the complexity of breeding heterozygote animals for 2 X-linked genes and 1 autosomal gene (ie, only 1 of 16 offspring mice are homozygote mutant or WT [wild type]) and the extensive requirement for genotyping of both breeders and offspring, homozygote mutant animals were used as breeders for this study (ie, *Nox1*^−/−^*/Nox2*^−/−^*/Nox4*^−/−^×*Nox1*^*−/y*^*/Nox2*^*−/y*^*/Nox4*^−/−^). Age-matched C57BL/6J mice maintained in a parallel colony were utilized as WT controls. Both sexes were used indistinctly. As a precaution, we made sure that the sex ratio was similar in the different experimental groups. Blood was collected by cardiac puncture according to local ethics regulations. For washed platelets, sodium citrate was used as an anticoagulant (0.5% w/v). Platelet-rich plasma was separated from whole blood by centrifugation (180*g*, 15 minutes), and platelets were separated from platelet-rich plasma by a second centrifugation step (600*g*, 10 minutes), in the presence of PGE1 (prostaglandin E1; 40 ng/mL) and indomethacin (10 μM). Platelets were resuspended in modified Tyrode buffer at a density of 2×10^8^ platelets/mL throughout the study. For whole-blood analyses (eg, thrombus formation), heparin- and D-phenylalanyl-L-prolyl-L-arginine chloromethyl ketone–anticoagulated whole blood was used (5 units/mL and 25 μM, respectively). All experiments performed with animals were in accordance with the German animal protection law and were performed after approval by the local authorities (Behörde für Gesundheit und Verbraucherschutz Freie und Hansestadt Hamburg, approval number 76/16).

### Electron Paramagnetic Resonance/Turbidimetry Assay

As described in our previous study,^5^ before adding stimuli, 200 µmol/L 1-hydroxy-3-methoxycarbonyl-2,2,5,5-tetramethylpyrrolidine (CMH), 5 µmol/L diethyldithiocarbamate, and 25 µmol/L deferoxamine were added to washed platelets (density adjusted to 2×10^8^ platelets/mL) with continuous stirring. After 1 minute, stimuli were delivered and aggregation was measured for 10 minutes by traditional turbidimetry as %absorbance decrease. After 10 minutes, 50 μL of platelet-free supernatant were read using an E-scan (Noxygen, Germany). Electron paramagnetic resonance spectra were recorded using the following electron paramagnetic resonance settings: center field, 3492.5 G; field sweep, 60 G; modulation amplitude, 2 G; sweep time, 10 s; number of scans, 10; microwave frequency, 9.39 GHz; microwave power, 20 mW; conversion time, 327.68 ms; time constant, 5242.88 ms. A calibration curve was obtained from standard CM^•^ diluted to concentrations of 0, 0.3, 1, 3, 10, and 30 µmol/L and utilized to estimate the CM^•^ concentration in the samples as described in Figure I in the Data Supplement. The CMH oxidation rate was obtained using the below formula:


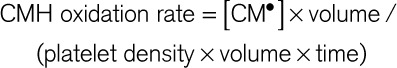


### Quantification of Intracellular cGMP by ELISA

Washed platelets were prepared as described above, and the levels of cGMP were quantified by competitive ELISA (No. KGE003; R&D Systems). Briefly, stimulation was performed in 200 μL of washed platelet suspensions (density adjusted to 2×10^8^ platelets/mL) with 10 μg/mL collagen or 0.25 u/mL thrombin for 20 minutes before cell lysis was obtained by 3 freeze/thaw cycles. Cell lysates were dispensed on 96-well microplate in triplicate; primary antibody and cGMP conjugate were added and then incubated for 3 hours. After 4 washes in washing buffer, the enzymatic substrate was added and incubated for 30 minutes at room temperature. After stop solution was added, the absorbance was measured at λ=450 nm.

### Thrombus Formation Under Physiological Flow Assay

Human and mouse blood was anticoagulated with 5 u/mL heparin and 40 µmol/L D-phenylalanyl-L-prolyl-L-arginine chloromethyl ketone and labeled with 1 μM 3,3′-dihexyloxacarbocyanine iodide for 10 minutes. Ibidi Vena8 Fluoro+ flow microchips and a Cellix Exigo pump were utilized to analyze thrombus formation in mouse whole blood under flow. Microchips were coated with Horm collagen (0.1 mg/mL) or fibrinogen (0.05 mg/mL). Thrombus formation was visualized by fluorescence microscopy at a shear rate of 200 or 1000 s^−1^. Surface coverage was analyzed using ImageJ 1.47v (Wayne Rasband, National Institutes of Health).

### Immunoblotting

Platelet suspensions prepared as described above were stimulated in the presence of 1 mmol/L EGTA and lysed by adding lysis buffer (12.5 mmol/L Tris, pH 8.3, 97 mmol/L glycine, 2% sodium dodecyl sulphate, 0.5% dithiothreitol, 10% glycerol, and 0.01% bromophenol blue). Platelet proteins were separated by SDS-PAGE, transferred to polyvinylidene difluoride membrane, and analyzed by immunoblotting for antiphosphotyrosine antibody (4G10, No. 05-321; Millipore), anti-PKC (protein kinase C) phospho-substrates (No. 2261; Cell Signaling Technology), anti-ERK (extracellular receptor kinase) antibodies (sc-94; Santa Cruz Biotechnology), anti-phospho-VASP (vasodilator-stimulated phosphoprotein; Ser 239; No. 3114; Cell Signaling Technology), anti-VASP (No. 3132; Cell Signaling Technology), or anti-actin (No. A5441; Merck Millipore). Densitometry was performed using ImageJ 1.47v (Wayne Rasband, National Institutes of Health). Data are presented as staining intensity of the target proteins with loading controls ERK or total VASP.

### Flow Cytometry

As described previously,^29^ washed platelets were stimulated with 1 u/mL thrombin or 5 μg/mL CRP-XL (cross-linked collagen-related peptide) and fixed in 1% w/v paraformaldehyde for 30 minutes. After diluting 1 in 10 in modified Tyrode buffer, PE-conjugated anti-integrin αIIbβ3 (No. M023-2, JON/A; EMFRET), PE-conjugated anti- CD62P (P-selectin; No. M130-2, Wug.E9; EMFRET), or FITC (fluorescein isothiocyanate)-annexin V (No. 556419; Pharmingen) were used to stain platelets, and fluorescence staining was assessed using a FACS Aria III flow cytometer (BD Biosciences).

### Pulmonary Thromboembolism Model

As described previously,^30^ mice were anesthetized by intraperitoneal administration of ketamine (120 mg/kg) and xylazine (16 mg/kg) in saline (10 mL/kg). Following laparotomy, Horm collagen (0.4 mg/kg) and epinephrine (60 μg/kg; Sigma-Aldrich) were slowly injected into the exposed inferior vena cava. After onset of respiratory arrest and while the heart was still beating, Evans blue dye (1% in saline) was retro-orbitally injected to assess lung perfusion. Time to death by pulmonary embolism was analyzed and utilized as a measure of susceptibility to thrombosis.

### Carotid Occlusion Model

Carotid occlusion was performed as described previously.^31^ Briefly, mice receiving 100 μL metamizol (200 mg/kg) subcutaneously for pain relief were anesthetized by inhalation of 4% isoflurane and continuously maintained at 1.5% isoflurane. A flow probe (Transonic Systems; TS420) was fitted around the exposed artery to monitor blood flow. Ferric chloride (FeCl_3_; 5% w/v) was applied topically to the exposed artery via a piece of filter paper (1×1.5 mm) to induce thrombus formation. After 3 minutes, the filter paper was removed while blood flow was continuously recorded until cessation (ie, occlusion) or alternatively up to 40 minutes in occlusion-free mice. Time to occlusion after FeCl_3_ challenge was analyzed and utilized as a measure of susceptibility to thrombosis.

### Tail-Tip Transection Assay

Under isoflurane-induced anesthesia (4% for induction and 1.5% for maintenance), animals were placed in prone position, and a 3-mm segment of the tail was amputated with a scalpel. The tail was immediately immersed in prewarmed isotonic saline at 37 °C. Bleeding time was measured manually.

### Thrombin Formation Assay

The procedure to measure thrombin generation using a fluorescent substrate for detection has been described previously.^32^ In short, 20 μL murine platelet-poor plasma (1:1 diluted in 20 μL of 20 mmol/L Tris buffer, pH 7.4) was incubated with tissue factor (6 pM) or kaolin (10 μg/mL) or no activator to a total volume of 60 μL containing 4 μM phospholipids (Thrombinoscope BV, Maastricht, the Netherlands), 16.6 mM Ca^2+^, and 2.5 mM fluorogenic substrate (ZGGR-AMC; Thrombinoscope BV). Thrombin parameters were calculated using Thrombinoscope software, version 3.0.0.29 (Thrombinoscope BV).

### Statistical Analysis

Data normality and homoscedasticity were tested with Shapiro-Wilk and Bartlett tests, respectively. For dual comparisons (ie, WT versus 3KO) of normal/homoscedastic data, statistical analysis was performed by unpaired Student *t* tests. Dual comparisons (ie, WT versus 3KO) of non-normal/nonhomoscedastic data were analyzed by nonparametric Mann-Whitney *U* test. One-way ANOVA with Bonferroni post test was used for multiple comparison tests after testing that data are normal and homoscedastic. The statistical software package GraphPad Prism, version 8.1.0, for Windows 64 bit was used. Results were expressed as the mean±SE (SEM). Differences were considered significant at *P*<0.05 (*), 0.01 (**), or 0.001 (***).

## Results

### Functional Characterization of 3KO Platelets In Vitro

**Figure 1.**
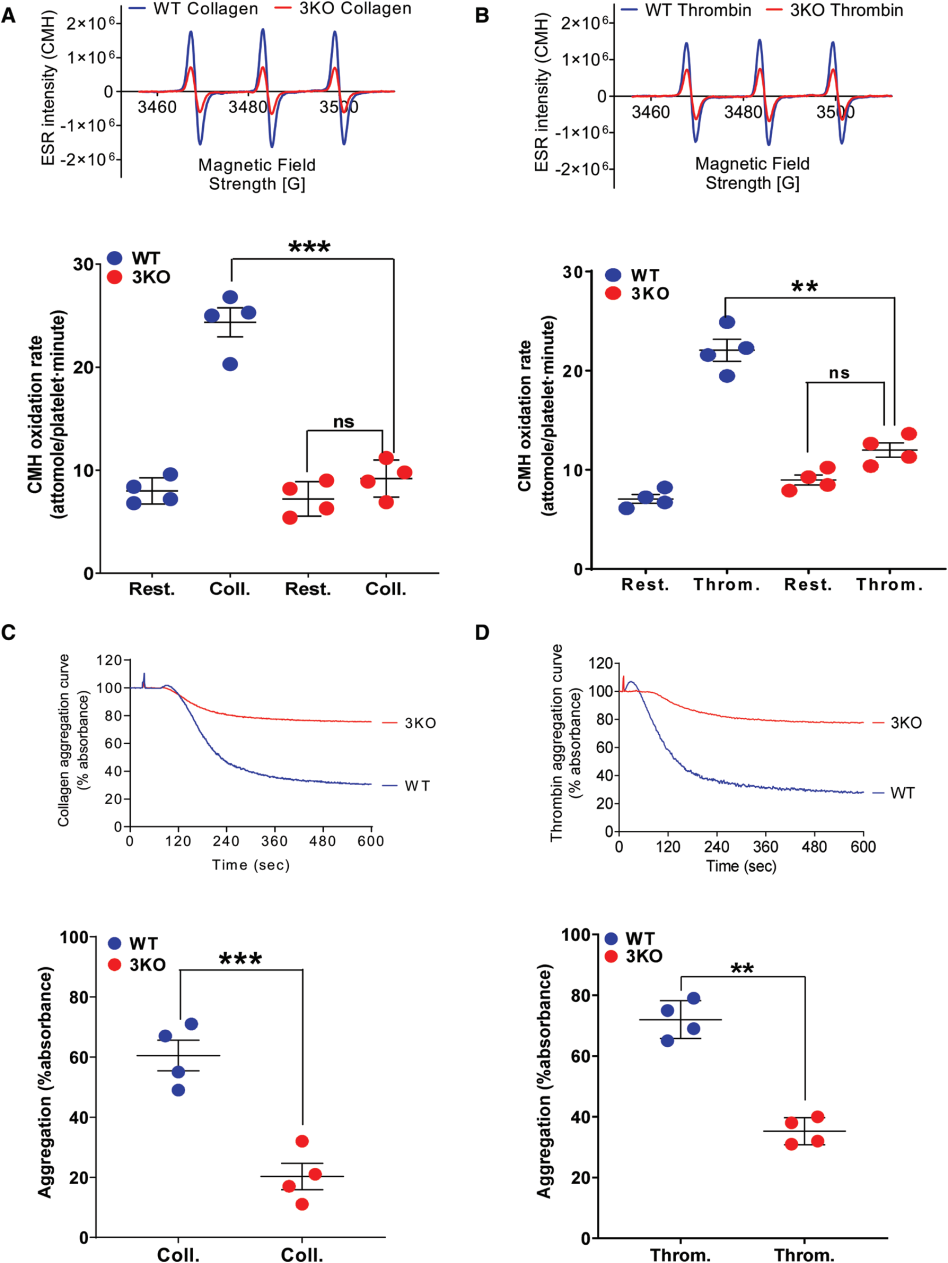
**Triple NOX (NADPH oxidase) 1, 2, and 4 deficiency attenuates superoxide radical generation (A and B) and platelet aggregation (C and D) in response to collagen or thrombin.** Superoxide radicals in response to 3 µg/mL fibrillar Horm collagen (**A**) or 0.1 unit/mL human thrombin (**B**) were measured by electron paramagnetic resonance spectroscopy (EPR). Representative EPR spectrograms (**top**) and quantitative analysis (**bottom**) are shown. Statistical analysis was tested by analyzed by 1-way ANOVA with Bonferroni post test. In parallel, aggregation in response to 3 µg/mL fibrillar Horm collagen (**C**) or 0.1 unit/mL human thrombin (**D**) was measured by turbidimetry. Representative aggregation traces (**top**) and quantitative analysis (**bottom**) are shown. Statistical analysis was tested by unpaired Student *t* test. 3KO indicates triple NOX knockout; CMH, 1-hydroxy-3-methoxycarbonyl-2,2,5,5-tetramethylpyrrolidine; Coll., collagen; ESR, electro spin resonance; ns, nonsignificant; Rest., resting; Throm., thrombin; and WT, wild type. ***P<0.05, **P<0.01, ***P<0.001.

**Figure 2.**
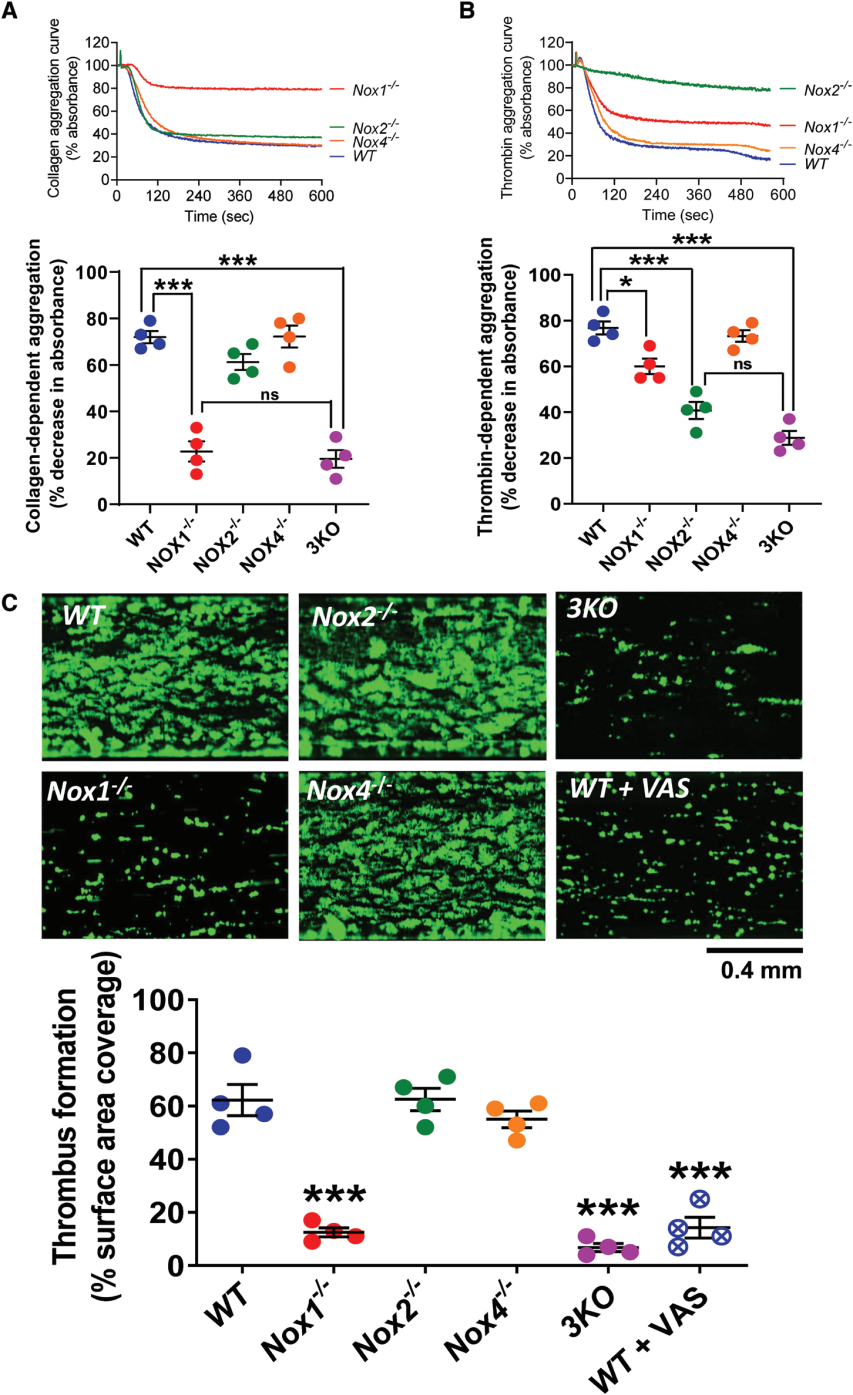
**Comparison of aggregation and thrombus formation responses of 3KO (triple NOX [NADPH oxidase] knockout) and single NOX knockout mice (Nox1^−/−^, Nox2^−/−^, and Nox4^−/−^).** Aggregation in response to 3 µg/mL fibrillar Horm collagen (**A**) or 0.1 unit/mL human thrombin (**B**) was measured by turbidimetry. Representative aggregation traces (**top**) and quantitative analysis (**bottom**) are shown. For thrombus formation under flow (**C**), heparin- and D-phenylalanyl-prolyl-arginyl chloromethyl ketone (PPACK)–anticoagulated whole blood was incubated with 1 μM 3,3′-dihexyloxacarbocyanine iodide (DiOC6) for 10 min, and thrombus formation was tested on collagen at intermediate shear stress (1000 s^−1^). Where indicated, WT (wild type) platelets were pretreated with 10 μM VAS2870 (VAS) for 10 min. Statistical analysis was performed using 1-way ANOVA with Bonferroni post test. ns indicates nonsignificant. *P<0.05, **P<0.01, ***P<0.001.

First, we confirmed the absence of hematological abnormalities of 3KO mice by full blood cell counts (Table I in the Data Supplement). After the levels of surface receptors in 3KO platelets were confirmed normal (Figure II in the Data Supplement), platelet function was studied using a combined electron paramagnetic resonance/turbidimetry assay that measures simultaneously superoxide radical formation and platelet aggregation (spin probe chemistry and calibration curve are shown in Figure I in the Data Supplement).^5^ Stimulation with collagen led to a significant increase in superoxide radical generation in WT controls (from 7.2±1.0 to 24.4±2.7 attomoles of CMH oxidized per platelet per minute) but not in 3KO mice (from 7.2±0.8 to 9.2±0.8 attomoles of CMH oxidized per platelet per minute; Figure 1A). Similarly, thrombin increased superoxide radical generation in WT platelets (from 7.2±1.0 to 22.1±3.9 attomoles of CMH oxidized per platelet per minute) but not in 3KO platelets (from 8.9±0.5 to 12.4±0.7 attomoles of CMH oxidized per platelet per minute; Figure 1B). Platelet aggregation in response to 3 μg/mL collagen (Figure 1C) or 0.1 unit/mL thrombin (Figure 1D) was ablated in 3KO compared with WT control mice. The comparison with single *NOX* gene knockouts (*Nox1*^−/−^, *Nox2*^−/−^, and *Nox4*^−/−^) showed that 3KO displays inhibition of collagen responses similar to *Nox1*^−/−^ mice (aggregation in Figure 2A and thrombus formation in Figure 2C) and thrombin responses similar to *Nox2*^−/−^ mice (aggregation in Figure 2B). Genetic deletion of Nox4 did not show any noticeable functional impairment (Figure 2A through 2C) and only a marginal reduction of superoxide radical output in response to collagen but not thrombin (Figure IIIA in the Data Supplement). The effect of triple NOX1, NOX2, and NOX4 deficiency can be recapitulated in human platelets with the pan NOX inhibitor VAS2870, which significantly inhibits agonist-induced superoxide radical output (Figure IIIB in the Data Supplement) and platelet aggregation in response to collagen (Figure IIIC in the Data Supplement) or thrombin (Figure IIID in the Data Supplement). Moreover, the pan inhibition with VAS2870 has similar effects on superoxide radical generation, platelet aggregation, and thrombus formation in C57BL6/J (the background of the 3KO mice) and BALB/c mice (Figure IV in the Data Supplement).

### Characterization of 3KO Platelet Signaling In Vitro

**Figure 3.**
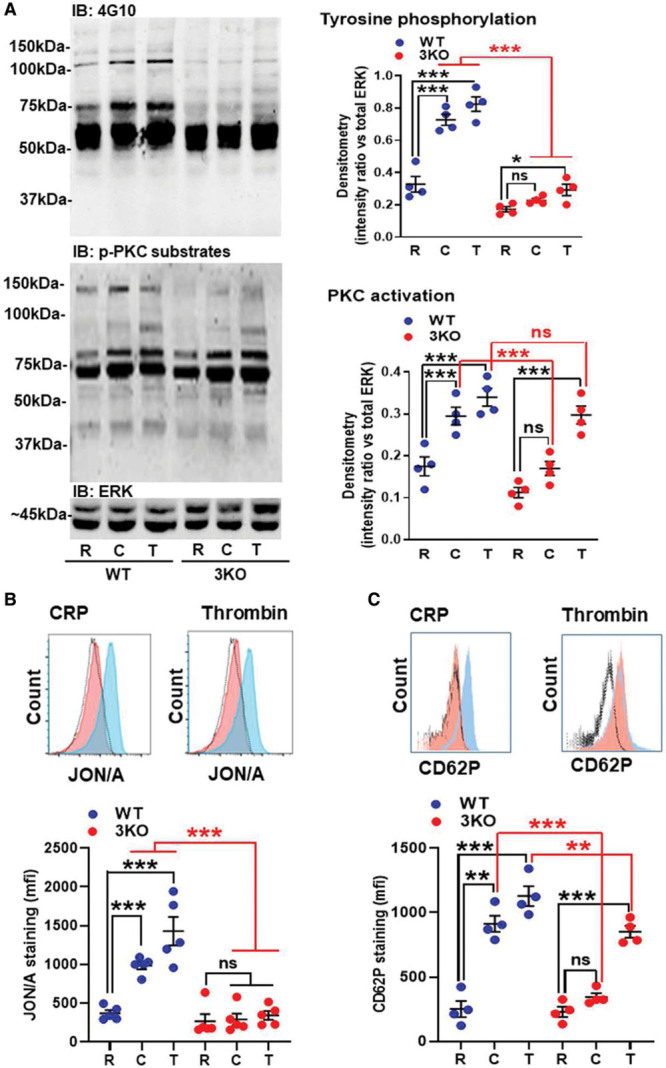
**Triple NOX (NADPH oxidase) 1, 2, and 4 deficiency impairs different components of the intracellular signaling of platelets.** The intracellular signaling of 3KO (triple NOX knockout) platelets was tested by immunoblotting protein extracts from resting (R), 10 μg/mL collagen (C)-stimulated, or 0.1 u/mL thrombin (T)-stimulated platelets (**A**). Tyrosine phosphorylation (**top**) and PKC (protein kinase C)-dependent phosphorylation (**bottom**) were tested. While anti-ERK (extracellular receptor kinase) was used as a loading control (**bottom**). Densitometry analysis was performed using ImageJ 1.47v (Wayne Rasband, National Institutes of Health) and is expressed as the ratio between the immunoreactivity intensity above 60 kDa for 4G10 and phosphorylated-PKC substrate blots and the intensity of ERK1/2 bands in the loading control blots for the same condition. Integrin αIIbβ3 activation and P-selectin surface expression (ie, a marker of degranulation) were tested by flow cytometry with JON/A (**B**) and anti-CD62P antibodies (**C**), respectively. Platelets were resting (R), activated by 5 μg/mL CRP-XL (C [cross-linked collagen-related peptide]) or activated with 1 u/mL thrombin (T). Statistical analysis was performed by 1-way ANOVA with Bonferroni post test with n=4 for **A** and **C** and n=5 for **B**.). CD62P indicates P-selectin; CRP, CRP-XL; ns, nonsignificant; p-PKC, phosphorylated PKC substrates; and WT, wild type. *P<0.05, **P<0.01, ***P<0.001.

**Figure 4.**
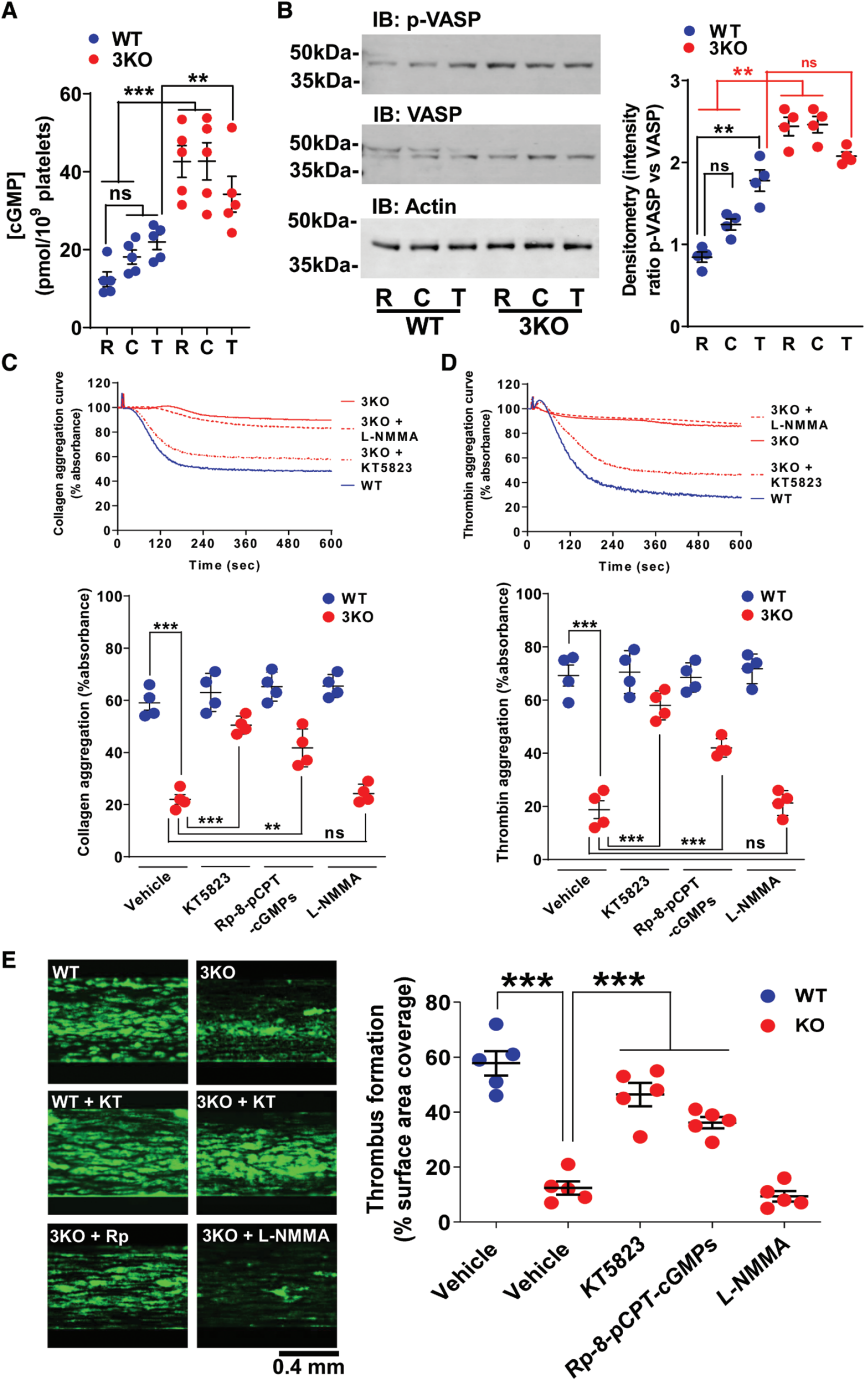
**Triple NOX (NADPH oxidase) 1, 2, and 4 deficiency attenuates platelet signaling by increasing cGMP levels.** For cGMP quantification (**A**), WT (wild type) and 3KO (triple NOX knockout) mouse washed platelets were left unstimulated (resting [R]) or stimulated with 10 μg/mL collagen (C) or 0.25 u/mL thrombin (T) for 20 min before cell lysis was obtained by 3 freeze/thaw cycles. cGMP was then quantified in the lysates following supplier instructions and expressed in pmol per 109 platelets. For immunoblotting (**B**), the same conditions as above were tested. Densitometry analysis was performed using ImageJ 1.47v (Wayne Rasband, National Institutes of Health) and is expressed as the ratio between the immunoreactivity intensity for p-VASP (Ser238) and total VASP (vasodilator-stimulated phosphoprotein). Aggregation in response to 3 µg/mL fibrillar platelet aggregation in response by Horm collagen (**C**) or 0.1 unit/mL human thrombin (**D**) and collagen-induced thrombus formation under physiological flow (1000 s^−1^) are shown in (**E**). Where indicated, platelets were preincubated with 1 μM KT5823 (KT), 5 μM Rp-8-pCPT-cGMPs (Rp), and 20 μM L-NG-monomethyl arginine (L-NMMA) or vehicle solution (0.1% dimethyl sulfoxide) for 30 min. For aggregation, representative traces (**top**) and quantitative analysis (**bottom**) are shown. For thrombus formation, representative pictures (**left**) and quantitative analysis (**right**) are shown. Data are analyzed by 1-way ANOVA with Bonferroni post test for **A** (n=5), **C** (n=4), **D** (n=4), and **E** (n=5), while nonparametric Kruskal-Wallis assay with Dunn post-test for **B** (n=4). ns indicates nonsignificant; p-VASP, phosphorylated VASP; and R, rest. *P<0.05, **P<0.01, ***P<0.001.

The activation of tyrosine kinase signaling cascades, which is fundamental for platelet activation,^33,34^ is significantly impaired in 3KO platelets stimulated by either collagen or thrombin, although a marginal response to thrombin remains (Figure 3A, top). In parallel, the activation of PKC, which is another pivotal component of the signaling of platelets,^35,36^ is lost in 3KO platelets in response to collagen but is preserved in response to thrombin (Figure 3A, middle). PKC activation was detected with antibodies against the phosphorylated classical PKC target sequence (ie, (R/K)-X-pS-hydhophobic-(R/K)),^37^ while tyrosine phosphorylation was tested using antibodies raised against phosphorylated tyrosine residues (clone 4G10). Multiband patterns shown in Figure 3A indicate multiple PKC- and tyrosine-phosphorylated proteins following platelet activation by either collagen or thrombin, respectively. Then, we assayed the activation of integrin αIIbβ3 by flow cytometry (Figure 3B). In these experiments, CRP-XL was used instead of collagen, which is a GP (platelet glycoprotein) VI–specific agonist compatible with flow cytometry (the fibrillar nature of collagen is problematic in these experiments).^38^ The levels of αIIbβ3 activation in response to CRP-XL (5 μg/mL) or thrombin (0.1 u/mL) were significantly reduced in 3KO compared with WT. In accordance, triple NOX deficiency impaired platelet adhesion on fibrinogen (the main ligand for integrin αIIbβ3), which was tested in 3,3′-dihexyloxacarbocyanine iodide–stained blood at low shear stress, 200 s^−1^ (Figure VA in the Data Supplement). In contrast, fibrinogen-dependent platelet adhesion under intermediate shear stress (1000 s^−1^) was not affected, which suggests that platelet adhesion in these conditions is driven by surface receptors other than integrin αIIbβ3 (eg, von Willebrand factor receptors; Figure VB in the Data Supplement). Combined PKC/Src kinase inhibition abolished platelet adhesion to fibrinogen at high shear stress (Figure VC in the Data Supplement). Next, we investigated platelet degranulation by flow cytometry using an anti–P-selectin antibody (ie, CD62P; Figure 3C). As for integrin αIIbβ3 activation, degranulation in response to collagen or thrombin was significantly impaired in 3KO platelets. As platelet responsiveness is negatively regulated by cyclic nucleotides,^39^ we analyzed the intracellular levels of cGMP in WT and 3KO platelets and found that the levels in 3KO platelets were consistently higher (from 12.3±1.8 to 42.6±4.8 pmol per 10^9^ platelets in resting conditions; Figure 4A). Despite an increasing trend, the stimulation of platelets with 10 μg/mL collagen or 0.25 u/mL thrombin for 20 minutes did not lead to a statistically significant change in intracellular cGMP. Pharmacological inhibition of NOXs with the pan inhibitor VAS2870 recapitulated in human platelets the increase in cGMP observed in 3KO mice, while inhibition of single NOX isoforms (either NOX1 or NOX2) with selective inhibitory peptides did not significantly affect intracellular cGMP (Figure VI in the Data Supplement).

As the adaptor molecule VASP is known to be phosphorylated by protein kinase G, we utilized an antibody that recognizes murine VASP when phosphorylated on serine 238 to assess the activation levels of PKG (protein kinase G; Figure 4B). 3KO mice displayed significantly higher levels of VASP phosphorylation compared with WT in resting and collagen-stimulated conditions, while following thrombin stimulation the level of VASP phosphorylation was not statistically different in the two mouse strains. Therefore, we assessed whether PKG inhibition can reverse the phenotype of 3KO mice. The PKG inhibitor KT5823 (1 μM) or Rp-8-pCPT-cGMPs (5 μM) significantly but partially reversed the inhibition of aggregation (Figure 4C and 4D) and thrombus formation (Figure 4E) of 3KO platelets, while inhibition of NOS (NO synthase) with L-NG-monomethyl arginine (20 μM) had no effect on 3KO platelet responses. PKG inhibition does not increase aggregation (Figure 4C and 4D) and thrombus formation (data not shown) in WT platelets.

### Characterization of Hemostasis and Thrombosis in 3KO Mice

**Figure 5.**
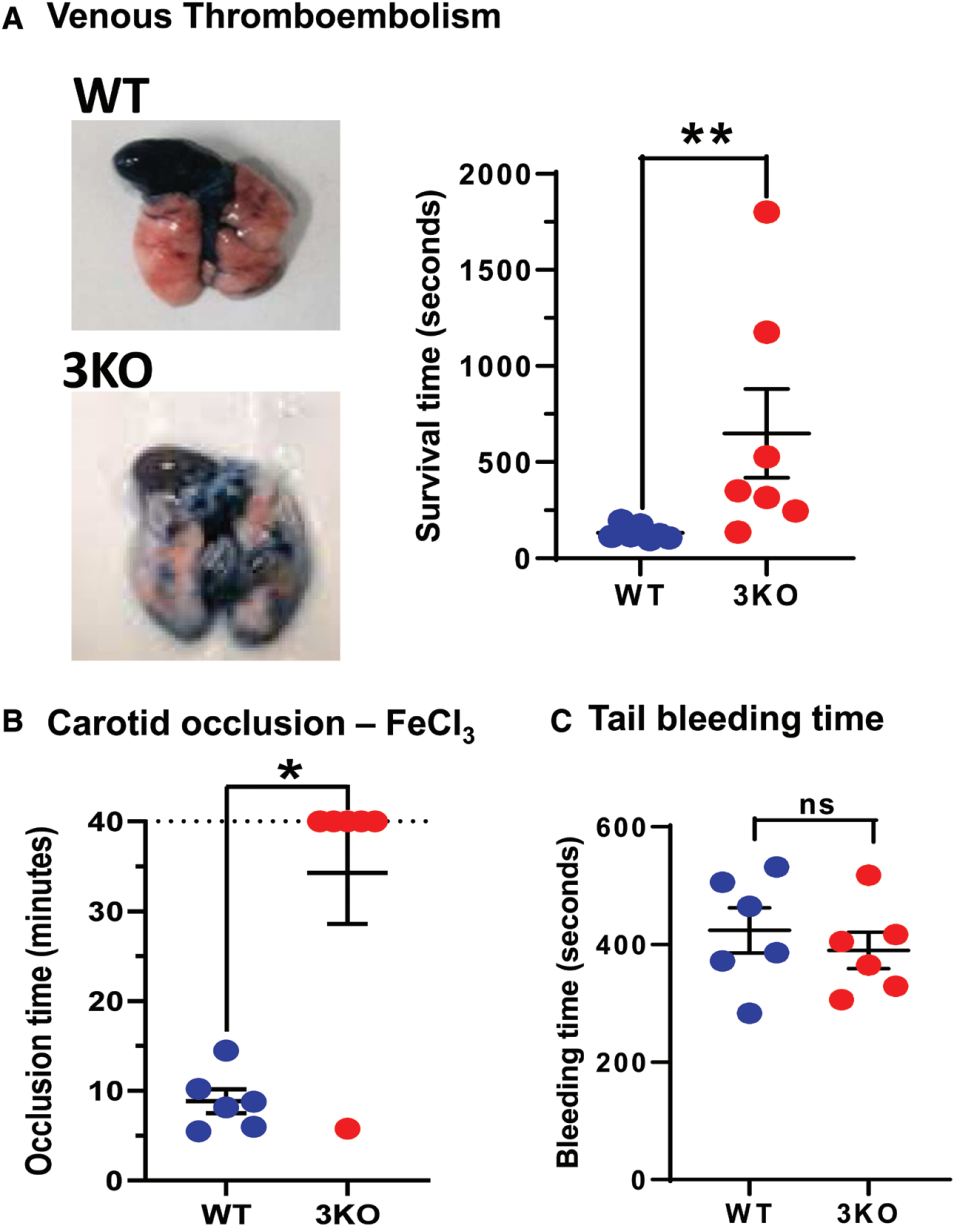
**Triple NOX (NADPH oxidase) 1, 2, and 4 deficiency protects against pulmonary thromboembolism (A) and arterial thrombosis (B) without affecting hemostasis (C) in vivo.** Pulmonary thromboembolism was induced by intravenous injection of a 0.4-mg/kg collagen and 60 μg/kg epinephrine into the vena cava (**A**). Time for thromboembolic death was measured and plotted (mean±SEM, n=7). Carotid occlusion was induced by application of 5% w/v ferric chloride (**B**). Occlusion times detected by Doppler ultrasound scanning for 3KO (triple NOX knockout) and WT (wild type) animals are plotted (mean±SEM, n=6). Bleeding time upon tail-tip transection is shown in **C** (mean±SEM plot, n=6). As data are not homoscedastic in **A** and **B** (ie, significantly different SD), statistical analysis was performed by nonparametric Mann-Whitney *U* test. Data in **C** are normally distributed and homoscedastic; therefore, parametric Student *t* test was used for the statistical analysis. ns indicates nonsignificant. *P<0.05, **P<0.01, ***P<0.001.

Next, we assessed whether the impairment of platelet activation observed in vitro had an impact on thrombosis in vivo. The susceptibility to thrombosis was tested in a collagen/epinephrine-induced lethal pulmonary thromboembolism model.^40,41^ As shown in Figure 5A, 3KO mice survive significantly longer after challenge compared with WT controls (332±80 versus 142±24 s, n=7, *P*=0.032). To confirm pulmonary embolism, we determined lung perfusion by intravenous administration of Evans blue dye (1% w/v). Perfused lung areas turned blue, whereas occluded parts remained a natural pinkish color. These data revealed improved perfusion of lung tissue in 3KO mice compared with WT mice. Next, we utilized a previously described model of arterial thrombosis based on the induction of carotid occlusion by FeCl_3_.^31^ As shown in Figure 5B, 3KO mice displayed a significant protection against carotid occlusion compared with WT controls (34.3±5.6 versus 8.8±1.3 minutes, n=6, *P*=0.035). Notably, the difference in carotid occlusion time between 3KO and WT mice is likely to be underestimated as the occlusion time limit of 40 minutes imposed by animal ethics was reached by 5 of 6 3KO mice, compared with 0 of 6 WT mice. To confirm that NOX deficiency in platelet rather than other cells was responsible for the reduction in thrombosis, we performed platelet transfer experiments as described previously.^42^ Thrombocytopenia was induced in WT mice by venous administration of the R300 antibody, and 12 hours later, platelets from either WT or 3KO were injected into the thrombocytopenic mice. Carotid occlusion assays were then performed as described. No thrombus formation was detected in thrombocytopenic mice receiving 3KO platelets, while abundant platelet deposition and thrombus formation were observed in mice receiving WT platelets (Figure VII in the Data Supplement). Finally, to understand the effect of NOX deficiency on the hemostatic response in vivo, bleeding times of 3KO mice were determined using tail-tip transection experiments. Bleeding time in 3KO mice was not increased compared with WT animals (390±48 versus 423±58 s, n=4; Figure 5C).

**Figure 6.**
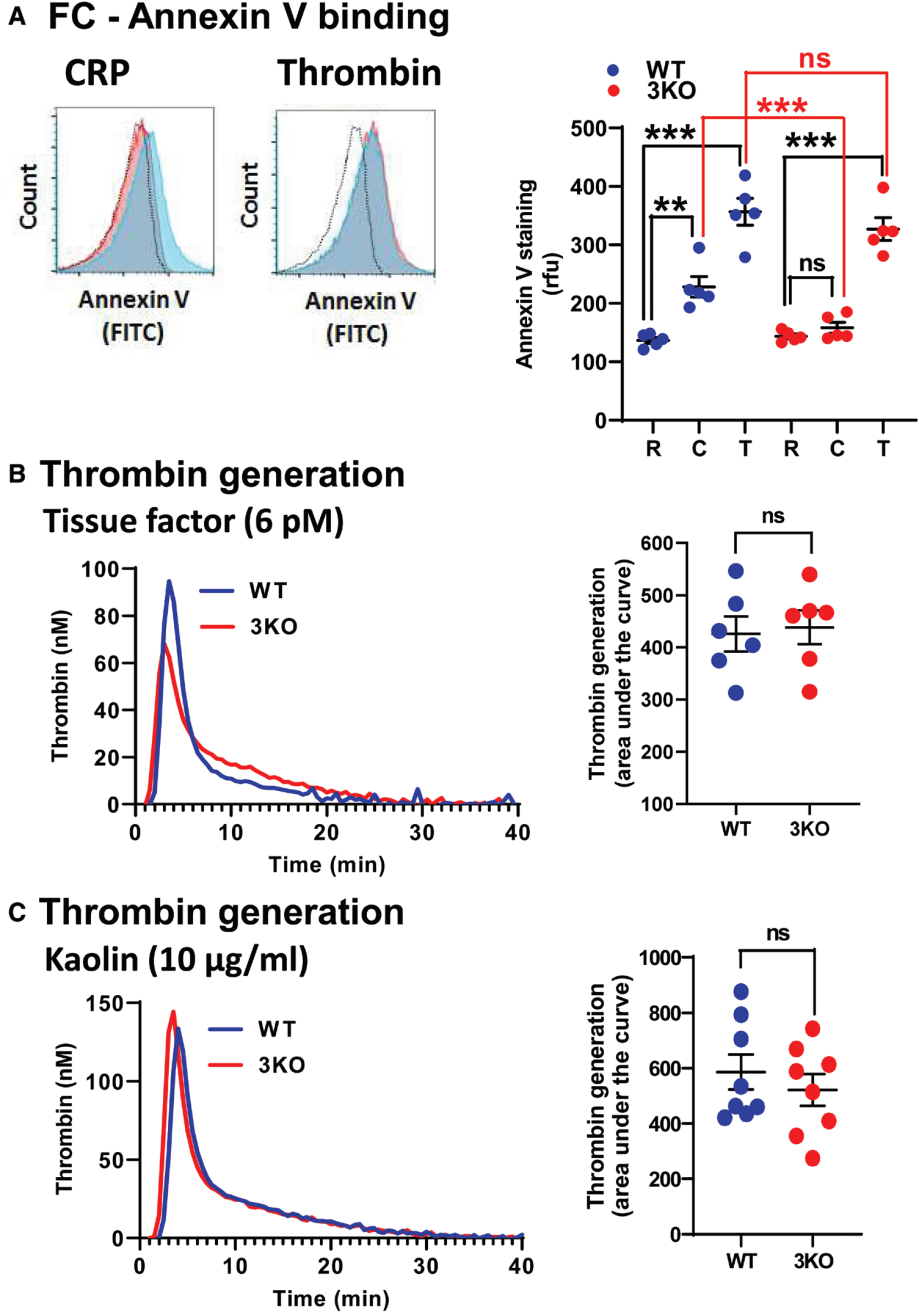
**Triple NOX (NADPH oxidase) 1, 2, and 4 deficiency does not affect the procoagulant activity of platelets (A) or coagulation response in plasma (B and C).** Phosphatidylserine externalization (**A**) was tested by flow cytometry. Platelets were resting (R) or activated by 5 μg/mL CRP-XL (C [cross-linked collagen-related peptide]) or activated by 1 u/mL thrombin (T) in the absence of shear stress for 40 min and then fixed (1% PFA) and labeled with FITC-annexin V. Representative histograms are shown in **top** (WT [wild type] in blue, 3KO [triple NOX knockout] in red, and unstimulated in dotted black), and statistical analysis is shown **bottom** (n=5). Statistical analysis was performed by 1-way ANOVA with Bonferroni post test. Thrombin generation in platelet-poor plasma in response to either 6 pM tissue factor (**B**) or 10 μg/mL kaolin (**C**) is shown (representative thrombin generation peaks in the **left**; mean±SEM for the area under the curve in the **right**; n=6 for tissue factor and n=8 for kaolin). For both **B** and **C**, the statistical analysis was performed by unpaired Student *t* test. CRP indicates CRP-XL; FC, flow cytometry; FITC, fluorescein isothiocyanate; and ns, nonsignificant. *P<0.05, **P<0.01, ***P<0.001.

To understand whether normal coagulation occurs normally in 3KO mice and sustains hemostasis, we first tested platelet phosphatidylserine (PS) externalization (which is a key procoagulatory response).^43^ PS externalization resulted impaired in 3KO compared with WT platelets only in response to collagen but not thrombin (Figure 6A), which may indicate differential roles for NOXs in collagen and thrombin signaling. In parallel, we tested whether the triple NOX deficiency has any effect on the coagulation cascade. For this reason, we analyzed the generation of thrombin in platelet-poor plasma in response to the activation of extrinsic (tissue factor) or intrinsic (kaolin) coagulation pathways (Figure 6B and 6C, respectively). No differences between 3KO and WT platelet-poor plasma were detected, suggesting that NOX deficiency has no impact on the coagulation cascade.

## Discussion

Despite observing ≈20% residual aggregation of platelets in 3KO mice, our data unequivocally suggest that NOXs are required for complete platelet aggregation by collagen or thrombin. This is in agreement with our recent study with single NOX1^−/−^ and NOX2^−/−^ knockout mice^5^ and other studies suggesting that either NOX1 or NOX2 is required for full platelet aggregation.^1–3,6,12^ This observation is also in agreement with a recent study showing that small molecules inhibiting NOX2 activation by Rac1 (Ras-related C3 botulinum toxin substrate 1) affect both collagen- and thrombin-dependent platelet responses.^14^ As Rac1 positively regulates both NOX1^44^ and NOX2,^45^ these data are in accordance with our results presented in this article.

Thrombus formation on collagen and platelet adhesion on fibrinogen were tested in whole blood flow assays, which is the most physiologically relevant ex vivo model of thrombosis.^46^ 3KO platelets displayed significantly impaired thrombus formation on collagen-coated surfaces at arterial shear stress (1000 s^−1^). This is in agreement with our data and previous studies showing a central role for NOXs in collagen signaling.^5–7,10,12,14,15,47^ The exact identity of the NOX family member(s) involved in GP VI signaling remains undetermined, with some studies suggesting NOX1^5,7^ and other NOX2.^6,47^ Our current data are clearly supporting the hypothesis that NOX1 is required for collagen signaling, while NOX2 is required for thrombin signaling (despite an involvement of NOX1). Interestingly, platelet adhesion on fibrinogen was inhibited at low (200 s^−1^) but not at intermediate shear stress (1000 s^−1^). As integrin αIIbβ3 is the main receptor for fibrinogen at low shear stress (<900 s^−1^),^48^ while GP 1b is required for adhesion at higher shear stress,^49^ our data suggest that only the former receptor is regulated by NOX-dependent superoxide radical formation. The dependence of integrin αIIbβ3 on NOX activity has previously been described in vitro.^2^ In agreement with these observations, our flow cytometry results using an antibody specific for active αIIbβ3 (ie, JON/A) suggest that the activation of this integrin is significantly impaired in the absence of NOXs in 3KO mice in response to either collagen or thrombin.

In this study, we provide information on the possible mechanism linking NOX activity to intracellular levels of cGMP, which negatively regulates platelets via activation of the protein kinase PKG. In 3KO platelets, we observed a statistically significant 4-fold increase in the intracellular levels of this negative regulator of platelet activity.^50^ Our data with PKG inhibitors (KT5823 and Rp-8-pCPT-cGMPs) suggest that the increase in cGMP and consequent activation of PKG play a key role in the loss of responsiveness of 3KO platelets. Nonetheless, alternative and parallel mechanisms of regulation also exist, as the rescue with PKG inhibitors is incomplete and single NOX isoform inhibition does not affect cGMP (possibly due to compensation between NOXs) while inhibiting platelet responses (at least partially). The existence of parallel NOX-dependent mechanisms regulating platelet responsiveness explains the different agonist selectivity of NOX1 and NOX2 ablation or inhibition (Figure 2). Contrarily to previous results showing that KT5823 could not rescue platelet inhibition induced by NO-releasing compounds,^51^ this inhibitor was effective in our experiments. This difference may be due to the different method used to increase cGMP (ie, NO-releasing agent in the abovementioned study versus NOX inhibition in our case) or to the different concentration of inhibitor used. In fact, we used 1 μM KT5823, which effectively inhibits PKG without affecting other kinases, while the abovementioned study describes the use of 5- to 10-μM KT5823, which are concentrations at which this inhibitor also affects PKC and presumably directly inhibits platelets. Importantly, a different PKG inhibitor (ie, Rp-8-pCTP-cGMPs) also significantly increased 3KO platelet responsiveness in our experiments, although to a lower level than KT5823. The difference between these two inhibitors could suggest parallel nonselective PKG-independent effects of KT5823. As NO is known to positively regulate soluble GC (guanylate cyclase) and increase cGMP levels,^39^ we tested an inhibitor of NOS (ie, L-NG-monomethyl arginine). The absence of any effect of NOS inhibition on 3KO platelet responsiveness suggests that the effect of NOXs on cGMP is NO independent, which is in line with reports of little or no expression of NOS in platelets.^52^ Alternative mechanisms of soluble GC activation by oxidant molecules described in other cell types are likely to underlie the effect of platelet NOXs on intracellular cGMP.^53,54^

We could not detect a decrease in the constitutive levels of superoxide generation for 3KO mice (Figure 1A and 1B). This could be due to sensitivity limitations in the electron paramagnetic resonance method or to the contribution of alternative enzymatic sources of superoxide anions (eg, respiration complexes in the mitochondria). It seems reasonable to assume that, due to their extremely short half-life, the cellular effects of superoxide anions heavily depend on the supramolecular interactions and cell compartmentalization of their enzymatic sources. Therefore, only NOX-dependent superoxide anions may be generated with the correct spatial-temporal patterns to affect cGMP levels.

In our immunostaining experiments, we detected some differences in the effect of triple NOX deficiency on collagen and thrombin signaling. Residual PKC activation and tyrosine phosphorylation responses were observed in platelets stimulated by thrombin but not collagen (Figure 3), which suggests differential involvement of NOXs in the signaling of collagen and thrombin (ie, more proximal to the stimulus for collagen and more distal for thrombin). In addition to the effect on cGMP and PKG described above, one plausible molecular mechanism underlying the effect of NOXs on collagen responses may involve the NOX-dependent oxidization and inhibition of SHP-2 (SH2 domain-containing protein tyrosine phosphatase-2), which has been suggested.^15,55–57^ The modulation of SHP2 in addition to PKG would explain more pronounced effects on collagen signaling, which is strongly affected by this protein phosphatase.^15,55^ The extensive inhibition of collagen receptor signaling accompanied by a more modest impairment of thrombin responses was mirrored in flow cytometry experiments for agonist-induced externalization of P-selectin (a marker of platelet degranulation; Figure 3C) and PS (a marker of platelet procoagulant activity; Figure 6A). In these experiments, triple NOX deficiency impaired the externalization of P-selectin and PS only in response to CRP-XL—a peptide stimulating the main collagen receptor GP VI.^38^ This reagent was used as the fibrillar nature of collagen makes it problematic to use in flow cytometry experiments. In contrast, both P-selectin and PS externalization in response to thrombin were not affected by NOX triple deficiency. This suggests that NOXs are intrinsically required for collagen signaling via the GP VI receptor, and without these enzymes, there is complete abolishment of any platelet response by this agonist. By contrast, thrombin remains able to induce a variety of platelet responses in addition to PKC activation and tyrosine phosphorylation, including degranulation (leading to P-selectin externalization) and PS exposure (which is critical for the procoagulant activity of platelets). These data suggest that NOXs act early in the signaling cascade of collagen, while they affect distal signaling events in the activation of platelets by thrombin (as described in the Graphic Abstract). This has been suggested previously.^7^

In our experiments, the platelet impairment caused by triple NOX genetic deletion led to a significant protection of 3KO animals from arterial thrombosis in carotid occlusion experiments and lung thromboembolism. Only few previous studies analyzed the role of NOXs in thrombotic responses in vivo, and again, there is lack of agreement in the literature. A previous study showed impairment of carotid occlusion in NOX2^−/−^ mice,^6^ while a more recent study suggested lack of effect of NOX2 genetic deletion on carotid occlusion.^19^ We recently published a study that agrees with the latter study and failed to detect any effect of NOX2 genetic deletion on carotid occlusion time, while NOX1 deletion was associated with a significant protection against this type of experimental thrombosis.^58^ Although it is difficult to conciliate these studies, differences in animals, conditions, and methods may be responsible for this discrepancy. In the current study, we provide definitive support for a role of NOXs as a family of enzymes in the regulation of thrombosis in vivo. In addition, we show that 3KO mice lacking platelet NOXs are protected against venous thrombosis in a model of lung thromboembolism (Figure 5A) while hemostasis is not affected (Figure 5C). Adoptive platelet experiments shown in Figure VII in the Data Supplement demonstrate that the effect on carotid occlusion depends on the silencing of NOXs in platelets rather than other blood or vascular cells. Taken together, our data support the use of current nonselective NOX inhibitors to prevent thrombosis without impairing the hemostatic response. Although non–platelet-specific NOX inhibition may have additional beneficial effects in cardiovascular disease (ie, reduction of inflammation and blood pressure^59,60^), considering the role of NOXs in the immune response,^4^ future studies will need to address the risk associated with NOX pharmacological inhibition, especially in long-term treatment studies both in experimental animals and human subjects.

## Acknowledgments

D. Vara, R.K. Mailer, A. Tarafdar, N. Wolska, S. Konrath, M. Spaeth, and M. Heestermans performed the experiments and analyzed the data. T. Renné and K. Schröder participated in experimental design, data analysis, and manuscript writing. G. Pula designed the study, analyzed the data, and wrote the manuscript.

## Sources of Funding

This work was funded by British Heart Foundation (PG/15/40/31522) and Alzheimer Research UK (ARUK-PG2017A-3) grants to G. Pula, European Research Council (ERC-StG-2012-311575_F-12) and Deutsche Forschungsgemeinschaft (DFG; CRC877 TP A11 and CRC841 TPB8) grants to T. Renné and DFG grants (CRC815 TPA1 and SFB834 TPA2) to K. Schröder. Nina Wolska was supported by an ETIUDA7 doctoral scholarship founded by the National Science Centre–Poland (2019/32/T/NZ3/00333).

## Disclosures

None.

## Supplementary Material



## References

[1] Begonja AJ Teichmann L Geiger J Gambaryan S Walter U Platelet regulation by NO/cGMP signaling and NAD(P)H oxidase-generated ROS. Blood Cells Mol Dis. 2006;36:166–170. doi: 10.1016/j.bcmd.2005.12.028 16469512 10.1016/j.bcmd.2005.12.028

[2] Begonja AJ Gambaryan S Geiger J Aktas B Pozgajova M Nieswandt B Walter U Platelet NAD(P)H-oxidase-generated ROS production regulates alphaIIbbeta3-integrin activation independent of the NO/cGMP pathway. Blood. 2005;106:2757–2760. doi: 10.1182/blood-2005-03-1047 15976180 10.1182/blood-2005-03-1047

[3] Chlopicki S Olszanecki R Janiszewski M Laurindo FR Panz T Miedzobrodzki J Functional role of NADPH oxidase in activation of platelets. Antioxid Redox Signal. 2004;6:691–698. doi: 10.1089/1523086041361640 15242549 10.1089/1523086041361640

[4] Carnevale R Loffredo L Sanguigni V Plebani A Rossi P Pignata C Martire B Finocchi A Pietrogrande MC Azzari C Different degrees of NADPH oxidase 2 regulation and in vivo platelet activation: lesson from chronic granulomatous disease. J Am Heart Assoc. 2014;3:e000920 doi: 10.1161/JAHA.114.000920 24973227 10.1161/JAHA.114.000920 PMC4309093

[5] Vara D Cifuentes-Pagano E Pagano PJ Pula G A novel combinatorial technique for simultaneous quantification of oxygen radicals and aggregation reveals unexpected redox patterns in the activation of platelets by different physiopathological stimuli. Haematologica. 2019;104:1879–1891. doi: 10.3324/haematol.2018.208819 30679320 10.3324/haematol.2018.208819 PMC6717585

[6] Delaney MK Kim K Estevez B Xu Z Stojanovic-Terpo A Shen B Ushio-Fukai M Cho J Du X Differential roles of the NADPH-oxidase 1 and 2 in platelet activation and thrombosis. Arterioscler Thromb Vasc Biol. 2016;36:846–854. doi: 10.1161/ATVBAHA.116.307308 26988594 10.1161/ATVBAHA.116.307308 PMC4850088

[7] Walsh TG Berndt MC Carrim N Cowman J Kenny D Metharom P The role of Nox1 and Nox2 in GPVI-dependent platelet activation and thrombus formation. Redox Biol. 2014;2:178–186. doi: 10.1016/j.redox.2013.12.023 24494191 10.1016/j.redox.2013.12.023 PMC3909778

[8] Magwenzi S Woodward C Wraith KS Aburima A Raslan Z Jones H McNeil C Wheatcroft S Yuldasheva N Febbriao M Oxidized LDL activates blood platelets through CD36/NOX2-mediated inhibition of the cGMP/protein kinase G signaling cascade. Blood. 2015;125:2693–2703. doi: 10.1182/blood-2014-05-574491 25710879 10.1182/blood-2014-05-574491 PMC4408294

[9] Calvieri C Tanzilli G Bartimoccia S Cangemi R Arrivi A Dominici M Cammisotto V Viceconte N Mangieri E Frati G Interplay between oxidative stress and platelet activation in coronary thrombus of STEMI patients. Antioxidants (Basel). 2018;7:83. 10.3390/antiox7070083 PMC6070897 29970802

[10] Carnevale R Bartimoccia S Nocella C Di Santo S Loffredo L Illuminati G Lombardi E Boz V Del Ben M De Marco L LDL oxidation by platelets propagates platelet activation via an oxidative stress-mediated mechanism. Atherosclerosis. 2014;237:108–116. doi: 10.1016/j.atherosclerosis.2014.08.041 25238217 10.1016/j.atherosclerosis.2014.08.041

[11] Abubaker AA Vara D Visconte C Eggleston I Torti M Canobbio I Pula G Amyloid peptide β1-42 induces integrin αIIbβ3 activation, platelet adhesion, and thrombus formation in a NADPH oxidase-dependent manner. Oxid Med Cell Longev. 2019;2019:1050476 doi: 10.1155/2019/1050476 31007831 10.1155/2019/1050476 PMC6441506

[12] Vara D Campanella M Pula G The novel NOX inhibitor 2-acetylphenothiazine impairs collagen-dependent thrombus formation in a GPVI-dependent manner. Br J Pharmacol. 2013;168:212–224. doi: 10.1111/j.1476-5381.2012.02130.x 22881838 10.1111/j.1476-5381.2012.02130.x PMC3570016

[13] Fuentes E Gibbins JM Holbrook LM Palomo I NADPH oxidase 2 (NOX2): a key target of oxidative stress-mediated platelet activation and thrombosis. Trends Cardiovasc Med. 2018;28:429–434. doi: 10.1016/j.tcm.2018.03.001 29661712 10.1016/j.tcm.2018.03.001

[14] Akbar H Duan X Piatt R Saleem S Davis AK Tandon NN Bergmeier W Zheng Y Small molecule targeting the Rac1-NOX2 interaction prevents collagen-related peptide and thrombin-induced reactive oxygen species generation and platelet activation. J Thromb Haemost. 2018;16:2083–2096. doi: 10.1111/jth.14240 30007118 10.1111/jth.14240 PMC6472274

[15] Wang SB Jang JY Chae YH Min JH Baek JY Kim M Park Y Hwang GS Ryu JS Chang TS Kaempferol suppresses collagen-induced platelet activation by inhibiting NADPH oxidase and protecting SHP-2 from oxidative inactivation. Free Radic Biol Med. 2015;83:41–53. doi: 10.1016/j.freeradbiomed.2015.01.018 25645952 10.1016/j.freeradbiomed.2015.01.018

[16] Ferroni P Vazzana N Riondino S Cuccurullo C Guadagni F Davì G Platelet function in health and disease: from molecular mechanisms, redox considerations to novel therapeutic opportunities. Antioxid Redox Signal. 2012;17:1447–1485. doi: 10.1089/ars.2011.4324 22458931 10.1089/ars.2011.4324

[17] Krötz F Sohn HY Pohl U Reactive oxygen species: players in the platelet game. Arterioscler Thromb Vasc Biol. 2004;24:1988–1996. doi: 10.1161/01.ATV.0000145574.90840.7d 15374851 10.1161/01.ATV.0000145574.90840.7d

[18] Krötz F Sohn HY Gloe T Zahler S Riexinger T Schiele TM Becker BF Theisen K Klauss V Pohl U NAD(P)H oxidase-dependent platelet superoxide anion release increases platelet recruitment. Blood. 2002;100:917–924. doi: 10.1182/blood.v100.3.917 12130503 10.1182/blood.v100.3.917

[19] Sonkar VK Kumar R Jensen M Wagner BA Sharathkumar AA Miller FJ Jr Fasano M Lentz SR Buettner GR Dayal S Nox2 NADPH oxidase is dispensable for platelet activation or arterial thrombosis in mice. Blood Adv. 2019;3:1272–1284. doi: 10.1182/bloodadvances.2018025569 30995985 10.1182/bloodadvances.2018025569 PMC6482355

[20] Pignatelli P Carnevale R Di Santo S Bartimoccia S Sanguigni V Lenti L Finocchi A Mendolicchio L Soresina AR Plebani A Inherited human gp91phox deficiency is associated with impaired isoprostane formation and platelet dysfunction. Arterioscler Thromb Vasc Biol. 2011;31:423–434. doi: 10.1161/ATVBAHA.110.217885 21071703 10.1161/ATVBAHA.110.217885

[21] El-Brolosy MA Stainier DYR Genetic compensation: a phenomenon in search of mechanisms. PLoS Genet. 2017;13:e1006780 doi: 10.1371/journal.pgen.1006780 28704371 10.1371/journal.pgen.1006780 PMC5509088

[22] Altenhöfer S Kleikers PW Radermacher KA Scheurer P Rob Hermans JJ Schiffers P Ho H Wingler K Schmidt HH The NOX toolbox: validating the role of NADPH oxidases in physiology and disease. Cell Mol Life Sci. 2012;69:2327–2343. doi: 10.1007/s00018-012-1010-9 22648375 10.1007/s00018-012-1010-9 PMC3383958

[23] Altenhöfer S Radermacher KA Kleikers PW Wingler K Schmidt HH Evolution of NADPH oxidase inhibitors: selectivity and mechanisms for target engagement. Antioxid Redox Signal. 2015;23:406–427. doi: 10.1089/ars.2013.5814 24383718 10.1089/ars.2013.5814 PMC4543484

[24] Hosseini E Ghasemzadeh M Atashibarg M Haghshenas M ROS scavenger, N-acetyl-l-cysteine and NOX specific inhibitor, VAS2870 reduce platelets apoptosis while enhancing their viability during storage. Transfusion. 2019;59:1333–1343. doi: 10.1111/trf.15114 30609081 10.1111/trf.15114

[25] Rezende F Löwe O Helfinger V Prior KK Walter M Zukunft S Fleming I Weissmann N Brandes RP Schröder K Unchanged NADPH oxidase activity in Nox1-Nox2-Nox4 triple knockout mice: what do NADPH-stimulated chemiluminescence assays really detect? Antioxid Redox Signal. 2016;24:392–399. doi: 10.1089/ars.2015.6314 25906178 10.1089/ars.2015.6314

[26] Gavazzi G Banfi B Deffert C Fiette L Schappi M Herrmann F Krause KH Decreased blood pressure in NOX1-deficient mice. FEBS Lett. 2006;580:497–504. doi: 10.1016/j.febslet.2005.12.049 16386251 10.1016/j.febslet.2005.12.049

[27] Pollock JD Williams DA Gifford MA Li LL Du X Fisherman J Orkin SH Doerschuk CM Dinauer MC Mouse model of X-linked chronic granulomatous disease, an inherited defect in phagocyte superoxide production. Nat Genet. 1995;9:202–209. doi: 10.1038/ng0295-202 7719350 10.1038/ng0295-202

[28] Zhang M Brewer AC Schröder K Santos CX Grieve DJ Wang M Anilkumar N Yu B Dong X Walker SJ NADPH oxidase-4 mediates protection against chronic load-induced stress in mouse hearts by enhancing angiogenesis. Proc Natl Acad Sci USA. 2010;107:18121–18126. doi: 10.1073/pnas.1009700107 20921387 10.1073/pnas.1009700107 PMC2964252

[29] Rochat S Alberio L Formaldehyde-fixation of platelets for flow cytometric measurement of phosphatidylserine exposure is feasible. Cytometry A. 2015;87:32–36. doi: 10.1002/cyto.a.22567 25266200 10.1002/cyto.a.22567

[30] Labberton L Kenne E Long AT Nickel KF Di Gennaro A Rigg RA Hernandez JS Butler L Maas C Stavrou EX Neutralizing blood-borne polyphosphate in vivo provides safe thromboprotection. Nat Commun. 2016;7:12616 doi: 10.1038/ncomms12616 27596064 10.1038/ncomms12616 PMC5025862

[31] Bonnard T Hagemeyer CE Ferric chloride-induced thrombosis mouse model on carotid artery and mesentery vessel. J Vis Exp. 2015;100:e52838. 10.3791/52838 PMC4544968 26167713

[32] Hemker HC Giesen P Al Dieri R Regnault V de Smedt E Wagenvoord R Lecompte T Béguin S Calibrated automated thrombin generation measurement in clotting plasma. Pathophysiol Haemost Thromb. 2003;33:4–15. doi: 10.1159/000071636 12853707 10.1159/000071636

[33] Stegner D Haining EJ Nieswandt B Targeting glycoprotein VI and the immunoreceptor tyrosine-based activation motif signaling pathway. Arterioscler Thromb Vasc Biol. 2014;34:1615–1620. doi: 10.1161/ATVBAHA.114.303408 24925975 10.1161/ATVBAHA.114.303408

[34] Senis YA Mazharian A Mori J Src family kinases: at the forefront of platelet activation. Blood. 2014;124:2013–2024. doi: 10.1182/blood-2014-01-453134 25115887 10.1182/blood-2014-01-453134 PMC4186533

[35] Gilio K Harper MT Cosemans JM Konopatskaya O Munnix IC Prinzen L Leitges M Liu Q Molkentin JD Heemskerk JW Functional divergence of platelet protein kinase C (PKC) isoforms in thrombus formation on collagen. J Biol Chem. 2010;285:23410–23419. doi: 10.1074/jbc.M110.136176 20479008 10.1074/jbc.M110.136176 PMC2906332

[36] Poole AW Pula G Hers I Crosby D Jones ML PKC-interacting proteins: from function to pharmacology. Trends Pharmacol Sci. 2004;25:528–535. doi: 10.1016/j.tips.2004.08.006 15380937 10.1016/j.tips.2004.08.006

[37] Nishikawa K Toker A Johannes FJ Songyang Z Cantley LC Determination of the specific substrate sequence motifs of protein kinase C isozymes. J Biol Chem. 1997;272:952–960. doi: 10.1074/jbc.272.2.952 8995387 10.1074/jbc.272.2.952

[38] Smethurst PA Onley DJ Jarvis GE O’Connor MN Knight CG Herr AB Ouwehand WH Farndale RW Structural basis for the platelet-collagen interaction: the smallest motif within collagen that recognizes and activates platelet glycoprotein VI contains two glycine-proline-hydroxyproline triplets. J Biol Chem. 2007;282:1296–1304. doi: 10.1074/jbc.M606479200 17085439 10.1074/jbc.M606479200

[39] Makhoul S Walter E Pagel O Walter U Sickmann A Gambaryan S Smolenski A Zahedi RP Jurk K Effects of the NO/soluble guanylate cyclase/cGMP system on the functions of human platelets. Nitric Oxide. 2018;76:71–80. doi: 10.1016/j.niox.2018.03.008 29550521 10.1016/j.niox.2018.03.008

[40] Nieswandt B Schulte V Bergmeier W Mokhtari-Nejad R Rackebrandt K Cazenave JP Ohlmann P Gachet C Zirngibl H Long-term antithrombotic protection by in vivo depletion of platelet glycoprotein VI in mice. J Exp Med. 2001;193:459–469. doi: 10.1084/jem.193.4.459 11181698 10.1084/jem.193.4.459 PMC2195902

[41] Renné T Pozgajová M Grüner S Schuh K Pauer HU Burfeind P Gailani D Nieswandt B Defective thrombus formation in mice lacking coagulation factor XII. J Exp Med. 2005;202:271–281. doi: 10.1084/jem.20050664 16009717 10.1084/jem.20050664 PMC2213000

[42] Cherpokova D Bender M Morowski M Kraft P Schuhmann MK Akbar SM Sultan CS Hughes CE Kleinschnitz C Stoll G SLAP/SLAP2 prevent excessive platelet (hem)ITAM signaling in thrombosis and ischemic stroke in mice. Blood. 2015;125:185–194. doi: 10.1182/blood-2014-06-580597 25301707 10.1182/blood-2014-06-580597 PMC4281827

[43] Lentz BR Exposure of platelet membrane phosphatidylserine regulates blood coagulation. Prog Lipid Res. 2003;42:423–438. doi: 10.1016/s0163-7827(03)00025-0 12814644 10.1016/s0163-7827(03)00025-0

[44] Cheng G Diebold BA Hughes Y Lambeth JD Nox1-dependent reactive oxygen generation is regulated by Rac1. J Biol Chem. 2006;281:17718–17726. doi: 10.1074/jbc.M512751200 16636067 10.1074/jbc.M512751200

[45] Koga H Terasawa H Nunoi H Takeshige K Inagaki F Sumimoto H Tetratricopeptide repeat (TPR) motifs of p67(phox) participate in interaction with the small GTPase Rac and activation of the phagocyte NADPH oxidase. J Biol Chem. 1999;274:25051–25060. doi: 10.1074/jbc.274.35.25051 10455184 10.1074/jbc.274.35.25051

[46] Nagy M van Geffen JP Stegner D Adams DJ Braun A de Witt SM Elvers M Geer MJ Kuijpers MJE Kunzelmann K Comparative analysis of microfluidics thrombus formation in multiple genetically modified mice: link to thrombosis and hemostasis. Front Cardiovasc Med. 2019;6:99 doi: 10.3389/fcvm.2019.00099 31417909 10.3389/fcvm.2019.00099 PMC6682619

[47] Cammisotto V Carnevale R Nocella C Stefanini L Bartimoccia S Coluccia A Silvestri R Pignatelli P Pastori D Violi F Nox2-mediated platelet activation by glycoprotein (GP) VI: effect of rivaroxaban alone and in combination with aspirin. Biochem Pharmacol. 2019;163:111–118. doi: 10.1016/j.bcp.2019.02.016 30771281 10.1016/j.bcp.2019.02.016

[48] Savage B Saldívar E Ruggeri ZM Initiation of platelet adhesion by arrest onto fibrinogen or translocation on von Willebrand factor. Cell. 1996;84:289–297. doi: 10.1016/s0092-8674(00)80983-6 8565074 10.1016/s0092-8674(00)80983-6

[49] Ruggeri ZM Platelet adhesion under flow. Microcirculation. 2009;16:58–83. doi: 10.1080/10739680802651477 19191170 10.1080/10739680802651477 PMC3057446

[50] Walter U Gambaryan S cGMP and cGMP-dependent protein kinase in platelets and blood cells. Handb Exp Pharmacol. 2009;191:533–548. 10.1007/978-3-540-68964-5_23 19089344

[51] Li Z Ajdic J Eigenthaler M Du X A predominant role for cAMP-dependent protein kinase in the cGMP-induced phosphorylation of vasodilator-stimulated phosphoprotein and platelet inhibition in humans. Blood. 2003;101:4423–4429. doi: 10.1182/blood-2002-10-3210 12576312 10.1182/blood-2002-10-3210

[52] Gambaryan S Tsikas D A review and discussion of platelet nitric oxide and nitric oxide synthase: do blood platelets produce nitric oxide from L-arginine or nitrite? Amino Acids. 2015;47:1779–1793. doi: 10.1007/s00726-015-1986-1 25929585 10.1007/s00726-015-1986-1

[53] Meurer S Pioch S Gross S Müller-Esterl W Reactive oxygen species induce tyrosine phosphorylation of and Src kinase recruitment to NO-sensitive guanylyl cyclase. J Biol Chem. 2005;280:33149–33156. doi: 10.1074/jbc.M507565200 16079134 10.1074/jbc.M507565200

[54] Tawa M Okamura T Soluble guanylate cyclase redox state under oxidative stress conditions in isolated monkey coronary arteries. Pharmacol Res Perspect. 2016;4:e00261 doi: 10.1002/prp2.261 27713826 10.1002/prp2.261 PMC5045941

[55] Jang JY Min JH Chae YH Baek JY Wang SB Park SJ Oh GT Lee SH Ho YS Chang TS Reactive oxygen species play a critical role in collagen-induced platelet activation via SHP-2 oxidation. Antioxid Redox Signal. 2014;20:2528–2540. doi: 10.1089/ars.2013.5337 24093153 10.1089/ars.2013.5337 PMC4025609

[56] Jang JY Min JH Wang SB Chae YH Baek JY Kim M Ryu JS Chang TS Resveratrol inhibits collagen-induced platelet stimulation through suppressing NADPH oxidase and oxidative inactivation of SH2 domain-containing protein tyrosine phosphatase-2. Free Radic Biol Med. 2015;89:842–851. doi: 10.1016/j.freeradbiomed.2015.10.413 26482867 10.1016/j.freeradbiomed.2015.10.413

[57] Qiao J Arthur JF Gardiner EE Andrews RK Zeng L Xu K Regulation of platelet activation and thrombus formation by reactive oxygen species. Redox Biol. 2018;14:126–130. doi: 10.1016/j.redox.2017.08.021 28888895 10.1016/j.redox.2017.08.021 PMC5596263

[58] Vara D Tarafdar A Celikag M Patinha D Gulacsy CE Hounslea E Warren Z Ferreira B Koeners MP Caggiano L NADPH oxidase 1 is a novel pharmacological target for the development of an antiplatelet drug without bleeding side effects [published online August 26, 2020]. FASEB J. doi: 10.1096/fj.202001086RRR 10.1096/fj.202001086RRR 32851720

[59] Knock GA NADPH oxidase in the vasculature: expression, regulation and signalling pathways; role in normal cardiovascular physiology and its dysregulation in hypertension. Free Radic Biol Med. 2019;145:385–427. doi: 10.1016/j.freeradbiomed.2019.09.029 31585207 10.1016/j.freeradbiomed.2019.09.029

[60] Hoffmann MH Griffiths HR The dual role of Reactive Oxygen Species in autoimmune and inflammatory diseases: evidence from preclinical models. Free Radic Biol Med. 2018;125:62–71. doi: 10.1016/j.freeradbiomed.2018.03.016 29550327 10.1016/j.freeradbiomed.2018.03.016

