# Supplementary figures and images for: NADPH Oxidases Are Required for Full Platelet Activation In Vitro and Thrombosis In Vivo but Dispensable for Plasma Coagulation and Hemostasis
Source: Arterioscler Thromb Vasc Biol. 2020 Dec 3;41(2):683–97. doi: 10.1161/ATVBAHA.120.315565 (PMC7837688; doi:10.1161/ATVBAHA.120.315565)

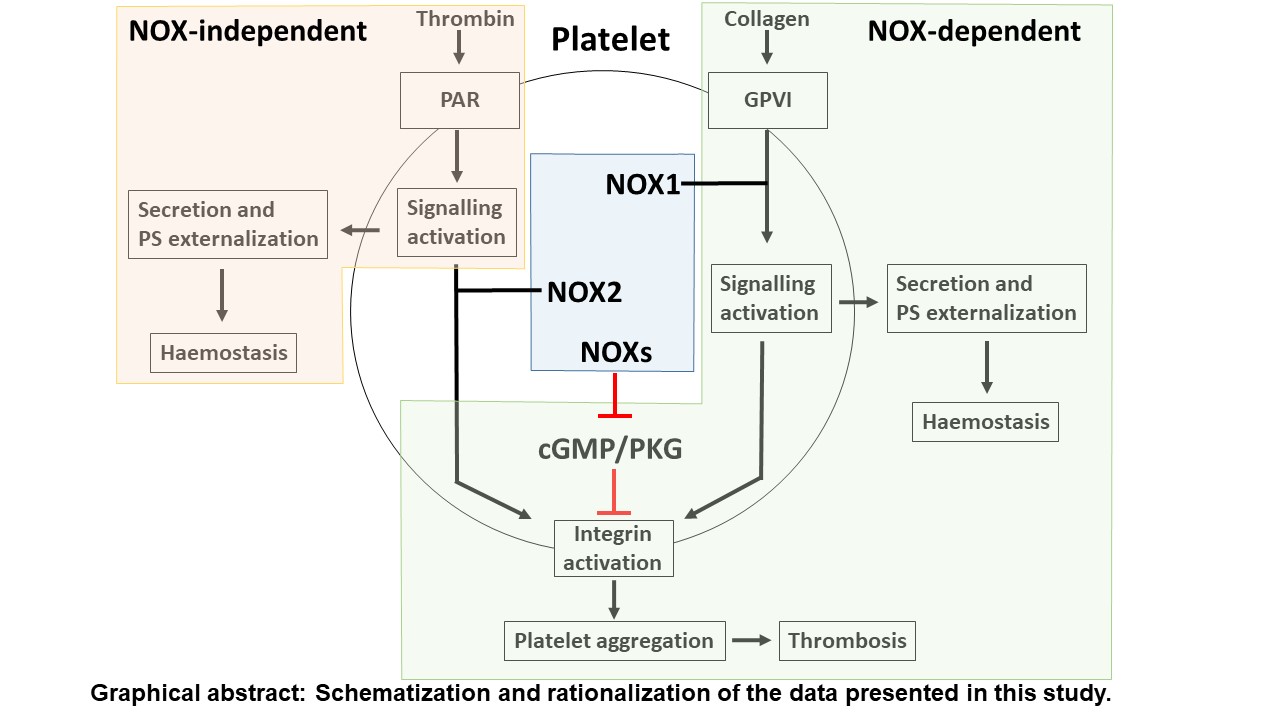

Supplement: Supplementary file 1 [file atv-41-683-s001.jpg]
