# Supplementary material for: NADPH Oxidases Are Required for Full Platelet Activation In Vitro and Thrombosis In Vivo but Dispensable for Plasma Coagulation and Hemostasis
Source: Arterioscler Thromb Vasc Biol. 2020 Dec 3;41(2):683–97. doi: 10.1161/ATVBAHA.120.315565 (PMC7837688; doi:10.1161/ATVBAHA.120.315565)
Supplement: Supplementary file 2 [file atv-41-683-s002.pdf]

SUPPLEMENTAL MATERIAL

NADPH oxidases are required for full platelet activation *in vitro* and thrombosis *in vivo*, but dispensable for plasma coagulation and haemostasis

Dina Vara [1], Reiner K. Mailer [2], Anuradha Tarafdar [3], Nina Wolska [2], Marco Heestermans [2], Sandra Konrath [2], Manuela Spaeth [4], Thomas Renné [2], Katrin Schröder [4], and Giordano Pula [2*]

[1]Institute of Biomedical and Clinical Science, University of Exeter Medical School, Exeter, UK. [2]Institute of Clinical Chemistry and Laboratory Medicine, University Medical Center Hamburg-Eppendorf, Hamburg, Germany. [3]Cancer Research UK Manchester Institute, University of Manchester, Manchester, UK. [4]Institute of Cardiovascular Physiology, Goethe-University, Frankfurt, Germany.

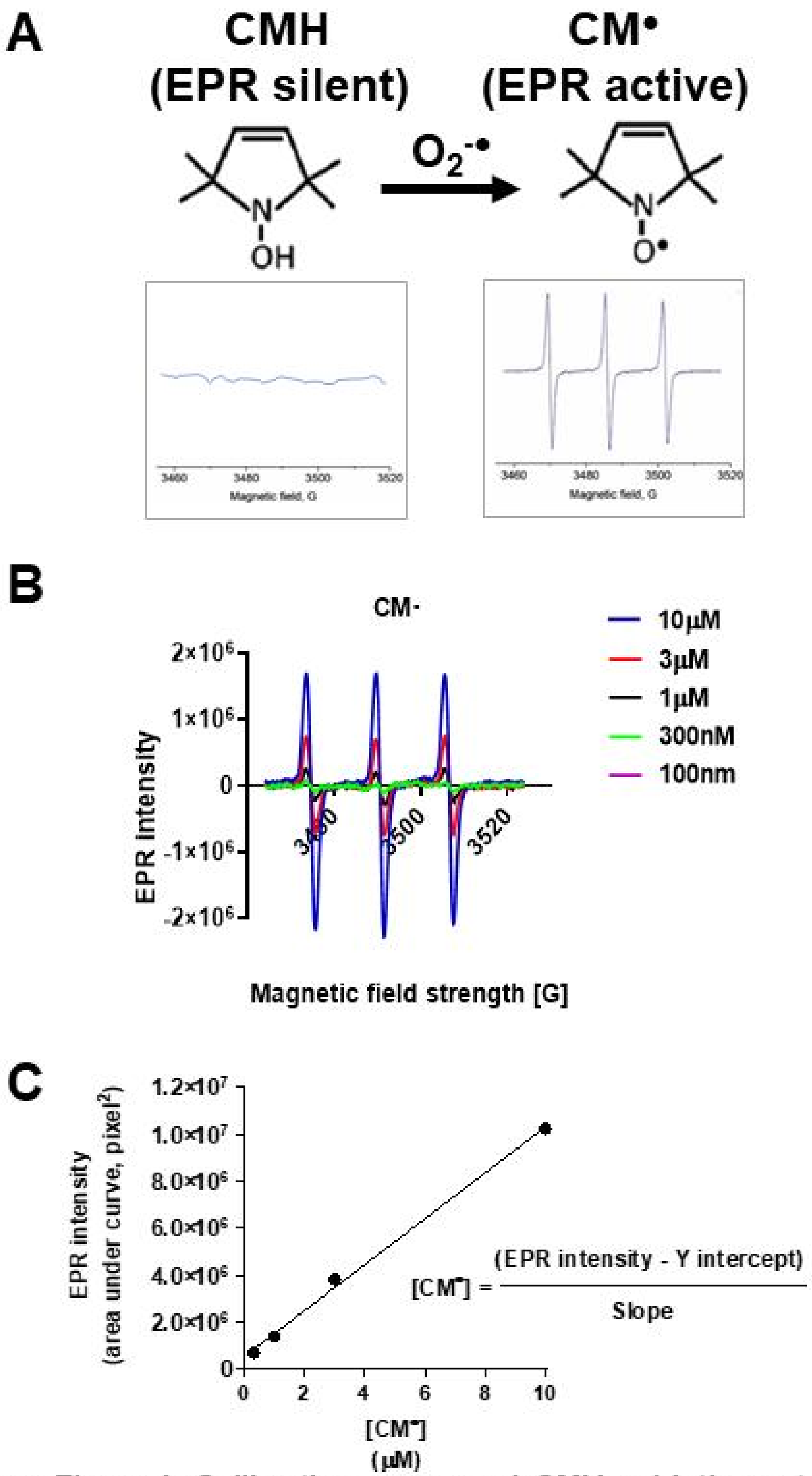

Supplementary Figure I: Calibration curve and CMH oxidation rate calculations. (A) Chemical structure and EPR properties of CMH and CM•. (B) Representative example of the concentration response curve for CM• (100nM to 10μM). (C) Calibration curve EPR intensity vs CM• concentration. This figure was modified from Vara et al., Haematologica 2019 Sep;104(9):1879-1891.

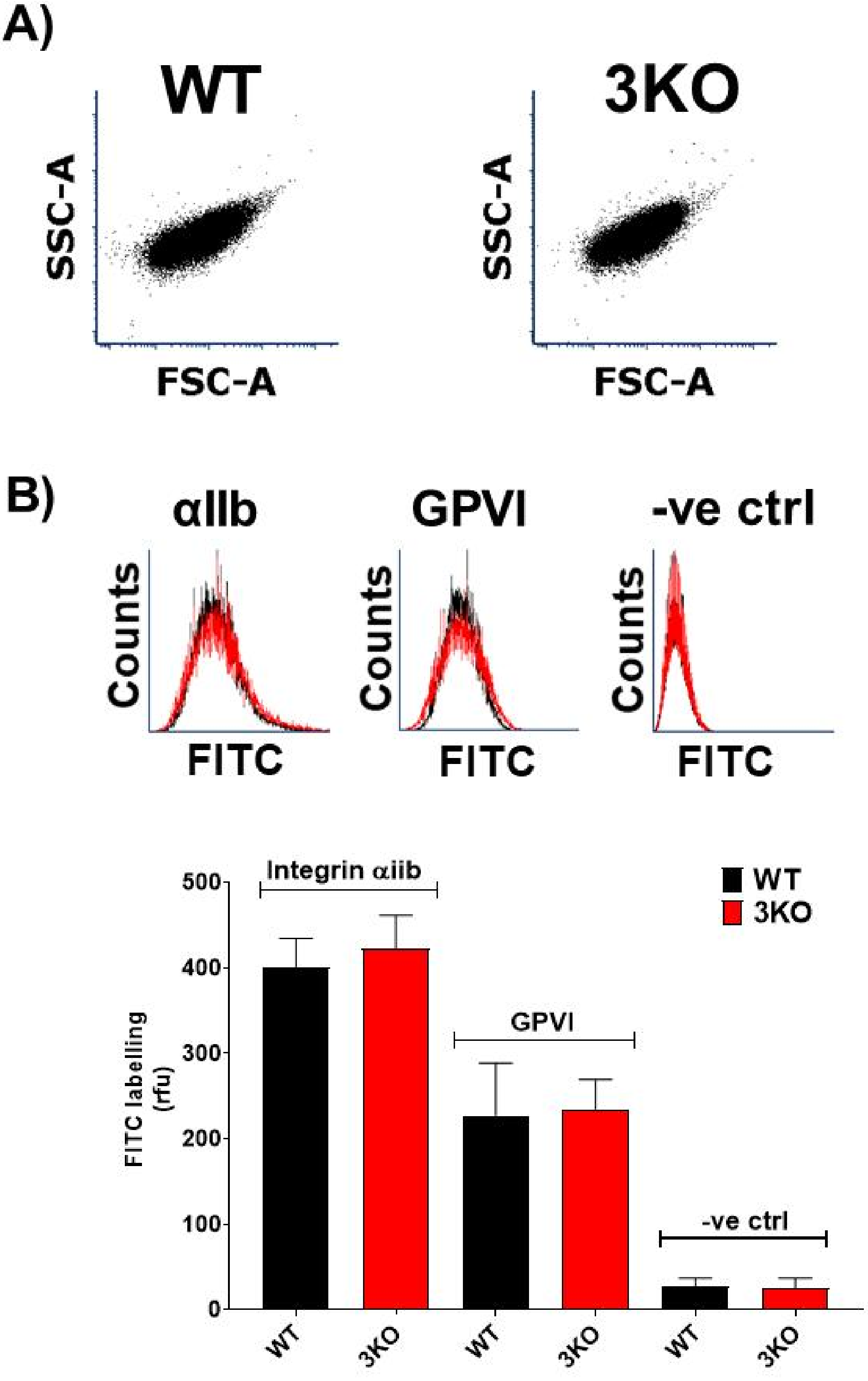

Supplementary figure ii. Flow cytometry characterisation of WT (C57Bl6/J) and 3KO platelets. Washed platelets were fixed in 1% w/v paraformaldehyde for 30 minutes. After diluting 1 in 10 in modified Tyrode's buffer, αIIbβ3 (#553847, Pharmingen) and GPVI (#PA5-20583, Thermo Fisher Scientifics) immunostaining was followed by fluorescent staining with secondary FITC-conjugated antibody. Negative control was secondary antibody alone. Representative histograms are shown in the top panel (WT in black, 3KO in red) and statistical analysis is shown in the bottom panel (n = 4). Statistical analysis was performed by one-way ANOVA with Bonferroni post-test and no significant difference was detected between WT and 3KO.

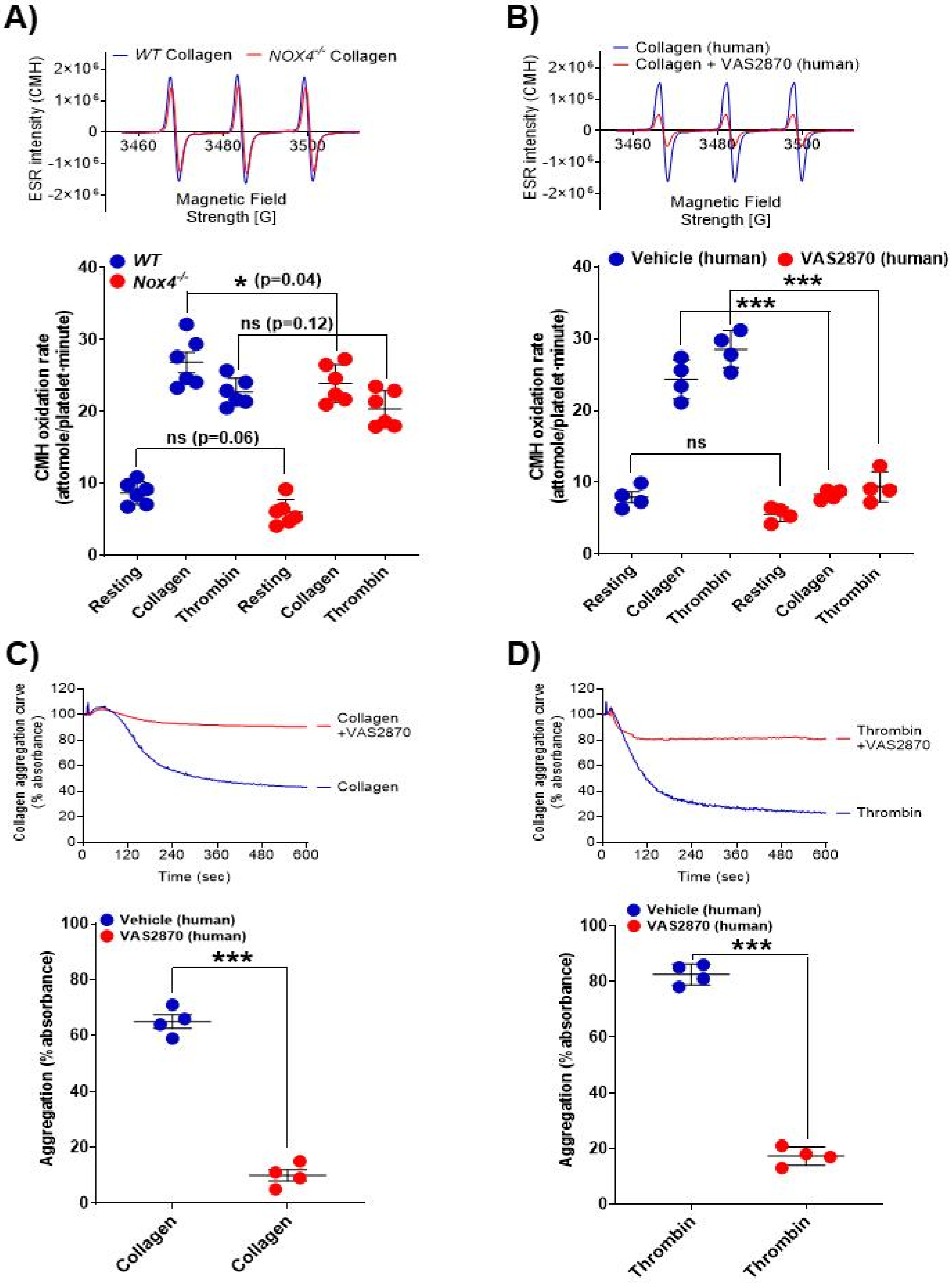

Supplementary Figure III. Analysis of mouse *Nox4*$^{-/-}$ mouse platelets and human platelets treated with the pan NOX inhibitor VAS2870. Superoxide radicals in response to 3 µg/ml fibrillar Horm collagen or 0.1 unit/ml human thrombin were measured in *Nox4*$^{-/-}$ mouse platelets (A) and human platelets (B) treated with 10 µM VAS2870 by electron paramagnetic resonance spectroscopy (EPR). Representative EPR spectrograms (top) and quantitative analysis (bottom) are shown. Statistical analysis was tested by analyzed by one-way ANOVA with Bonferroni post-test (*** for P<0.05, ** for P<0.01, *** for P<0.001, ns for non-significant). In parallel, aggregation in response to 3 µg/ml fibrillar Horm collagen (C) or 0.1 unit/ml human thrombin (D) were measured by turbidimetry. Representative aggregation traces (top) and quantitative analysis (bottom) are shown. Statistical analysis was tested by Student's t-test (for dual comparisons) and one-way ANOVA with Bonferroni post-test for multiple comparisons (*** for P<0.05, ** for P<0.01, *** for P<0.001, ns for non-significant).

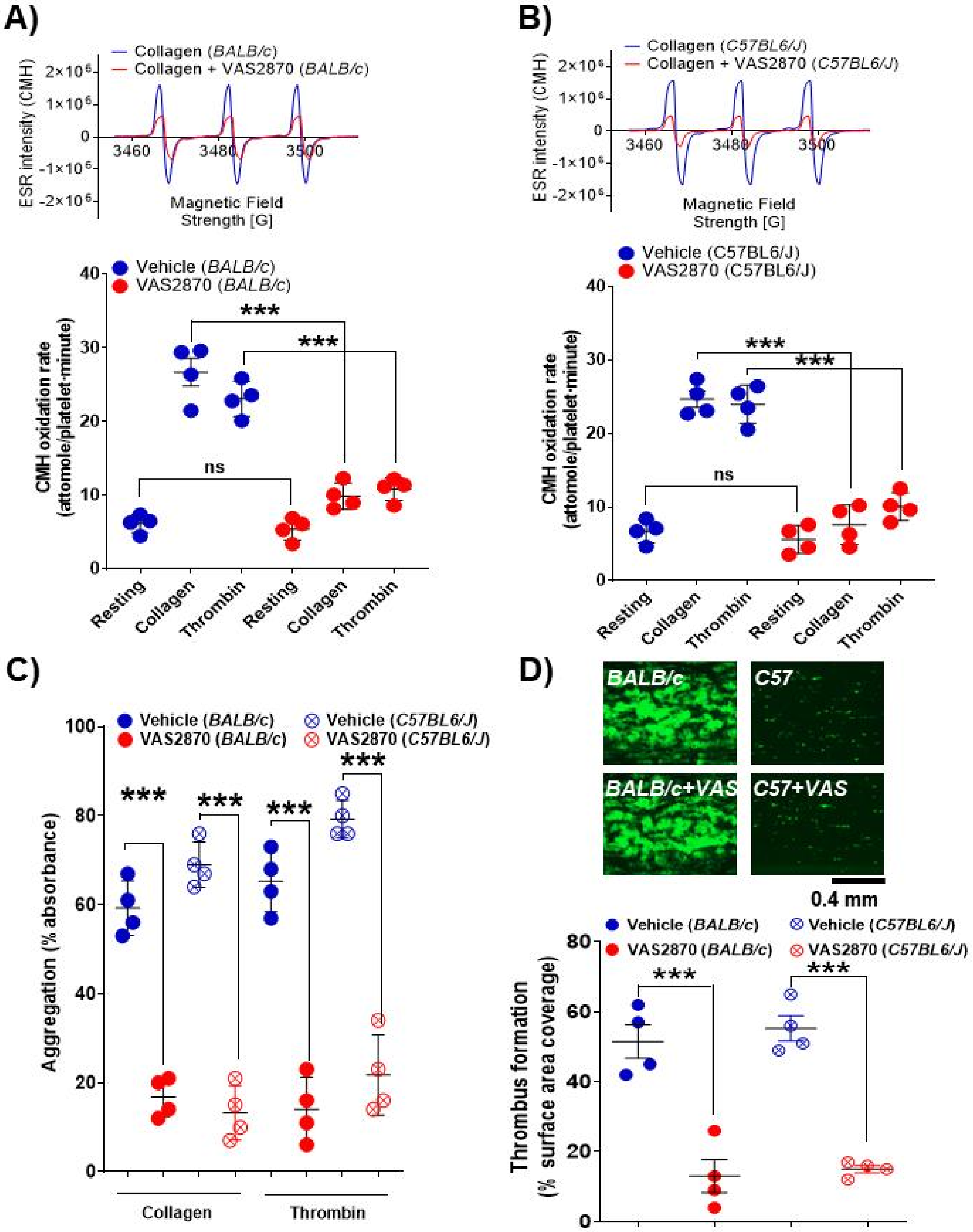

Supplementary Figure IV. Analysis of mouse *BALB/c* and *C57BL6/J* mouse platelets treated with the pan NOX inhibitor VAS2870. Superoxide radicals in response to 3 µg/ml fibrillar Horm collagen or 0.1 unit/ml human thrombin were measured in *BALB/c* (A) and *C57BL6/J* (B) mouse platelets treated with 10 µM VAS2870 by electron paramagnetic resonance spectroscopy (EPR). Representative EPR spectrograms (top) and quantitative analysis (bottom) are shown. In parallel, aggregation in response to 3 µg/ml fibrillar Horm collagen or 0.1 unit/ml human thrombin was measured by turbidimetry in *BALB/c* and *C57BL6/J* platelets treated with 10 µM VAS2870 (C). Collagen-dependent thrombus formation under flow conditions (1,000 sec^{-1}) was also tested in *BALB/c* and *C57BL6/J* platelets treated with 10 µM VAS2870. Statistical analysis was tested by Student's t-test (for dual comparisons) and one-way ANOVA with Bonferroni post-test for multiple comparisons (*** for $P<0.05$, ** for $P<0.01$, *** for $P<0.001$, ns for non-significant, n=4).

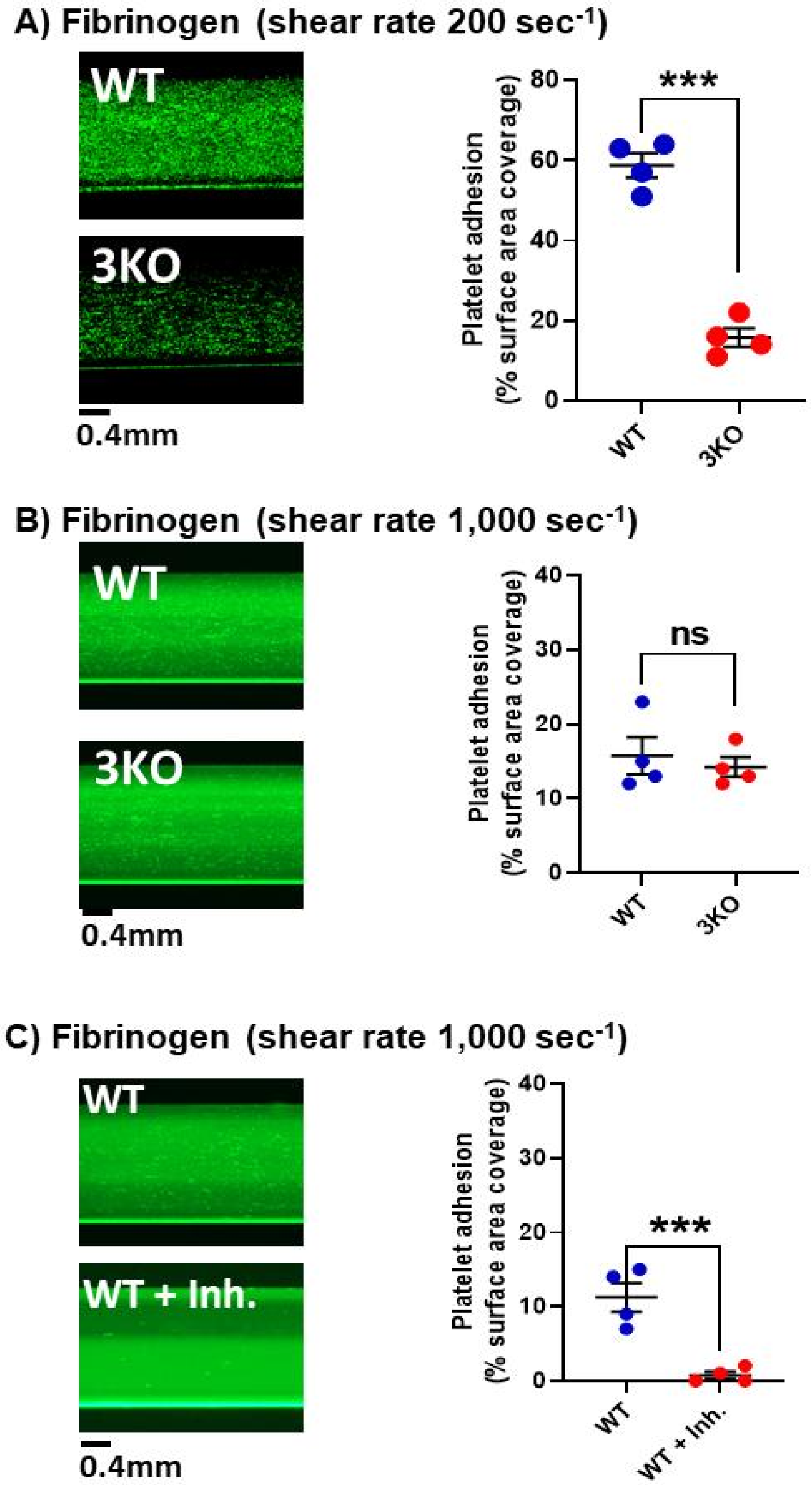

Supplementary Figure V. Platelet adhesion to fibrinogen in whole blood under shear stress conditions. Heparin- and PPACK-anticoagulated blood stained with DiOC6 was flowed on fibrinogen at low shear stress (200 sec-1) (A) or on fibrinogen at intermediate shear stress (1,000 sec^{-1}) (B). 3KO (red) and WT (blue) were compared. WT blood was also tested following treatment with the PKC/Src inhibitor cocktail (20 µM PP2 + 20 nM bisindolylmaleimide I) (C), which proved that platelet inhibition abolishes fibrinogen adhesion response at 1,000 sec^{-1} shear stress. Representative examples are in left panels, while statistical analysis performed by Student's t-test is shown on the right (* for P<0.05, ** for P<0.01, *** for P<0.001 ns for non-significant, n = 4).

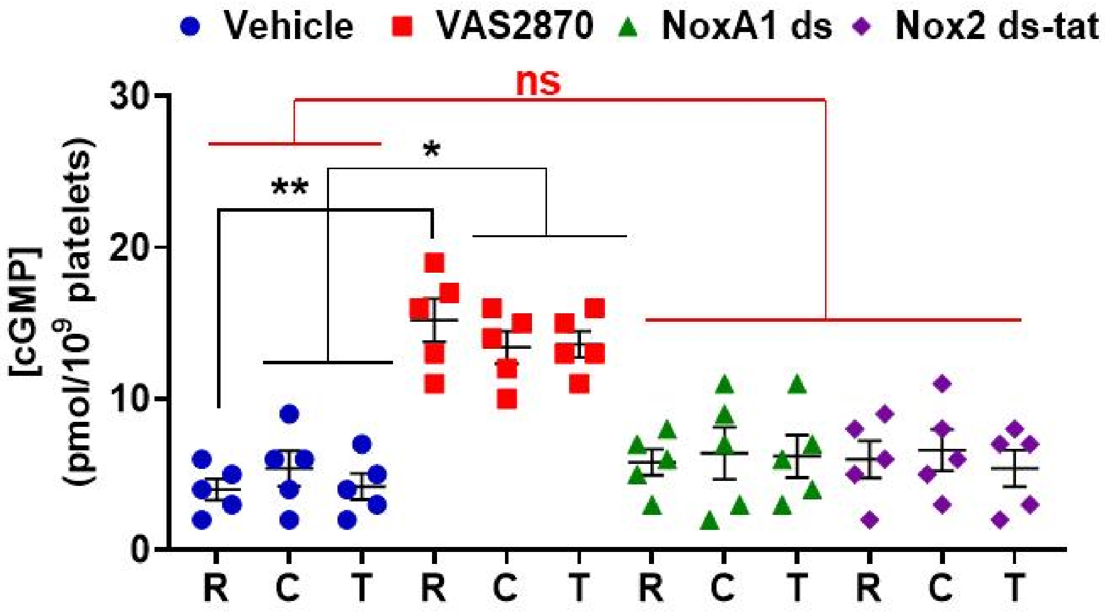

Supplementary Figure VI. NOX inhibition in human platelets increases cGMP levels. Human washed platelets were treated for 20 minutes with vehicle solution (0.1% DMSO), 10 µM VAS2870, 10 µM NoxA1 ds or 10 µM Nox2 ds-tat. Then platelets (were left unstimulated (abbreviated R) or stimulated with 10 µg/ml collagen (abbreviated C) or 0.25 u/ml thrombin (abbreviated T) for 20 minutes before cell lysis was obtained by 3 freeze/thaw cycles. cGMP was then quantified in the lysates following supplier instructions and expressed in pmol per 10^9 platelets. Data were analysed by one-way ANOVA with Bonferroni post-test (n =5) (* for P<0.05, ** for P<0.01, *** for P<0.001, ns for non-significant).

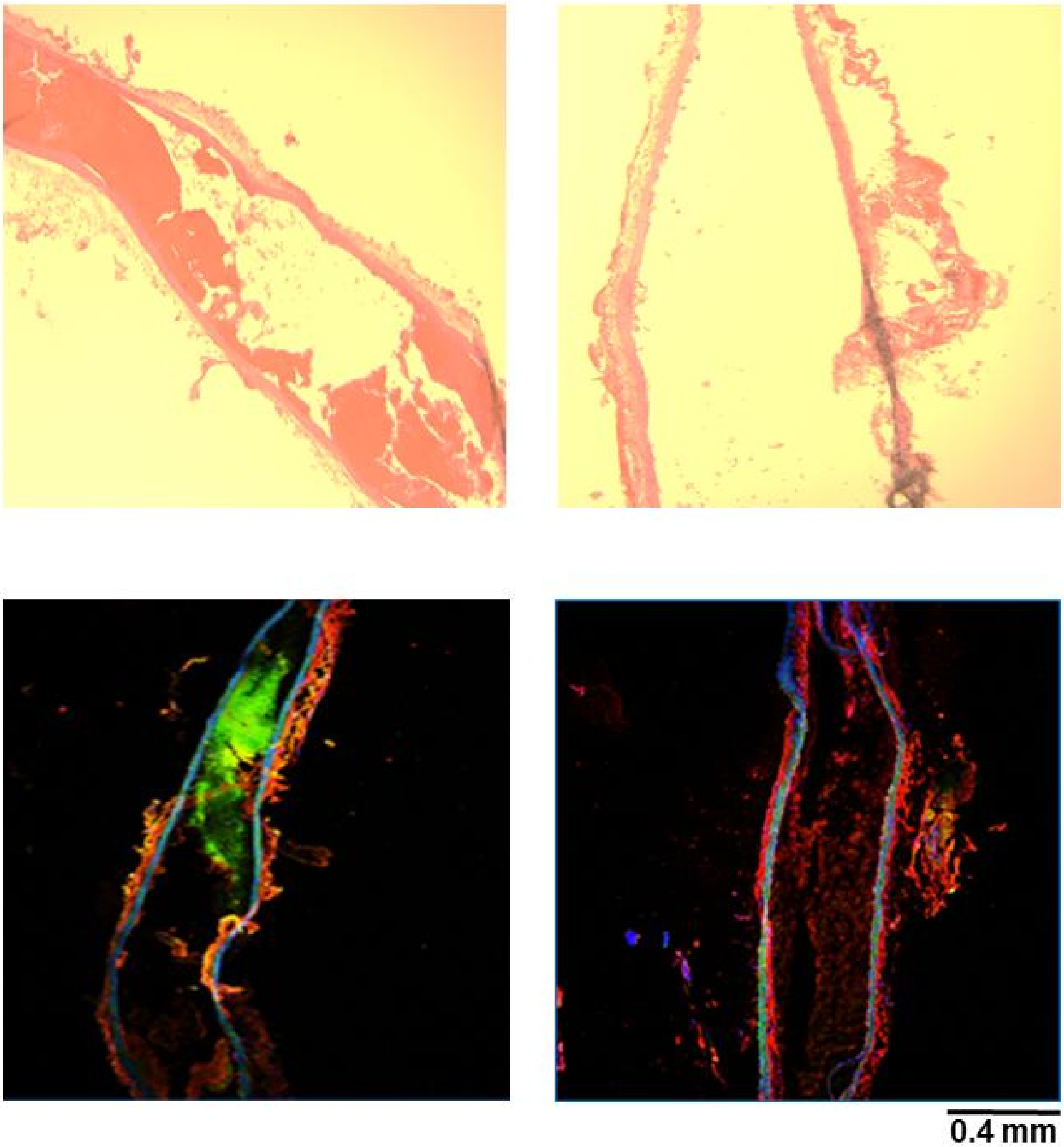

Supplementary Figure VII. Platelet replacement experiments and carotid occlusion experiments. Platelet depletion was induced in WT mice by IV injection of the anti-GPIbα antibody R300 (1 µg/g body weight). 12 hours after antibody injection, 6 x 10^8 platelets from either WT (A and C) or 3KO (B and D) ice were IV injected into thrombocytopenic mice (thrombocytopenia was confirmed by blood platelet counting). Carotid occlusion was induced as described in the manuscript ($FeCl_3$, 5% w/v). Carotids were explanted 40 minutes after ferric chloride treatment. Carotid were stained with traditional hematoxylin-eosin method (A-B). Alternatively, explanted carotids were immunostained (C-D) with antibodies against CD41 (platelet marker, ABCAM ab63983 1:50 + Alexa488 secondary (green)), CD31 (endothelial marker, Santa Cruz Biotechnologies sc-376764 1:50 + Alexa594 secondary (red)) and DAPI (nuclear stain, 1 µg/ml (blue)). Platelet deposited as thrombi are visible in mice that received WT platelets. The images are representative of experiments on 8 different animals.

	Normal range (Min)	Normal range (Max)	3KO	WT	P value (n=6)
RBC (M/µL)	**3.57**	**15.2**	**10.766**	**12.784**	**0.37**
HGB (g/dL)	**6.1**	**21.7**	**15.7**	**18.66**	**0.37**
HCT (%)	**16.7**	**69.8**	**64.2**	**78.26**	**0.30**
MCV (fL)	**39**	**90.8**	**59.58**	**62.92**	**0.31**
MCH (pg)	**12.6**	**31**	**14.56**	**14.88**	**0.72**
MCHC (g/dL)	**27**	**37.6**	**24.6**	**23.74**	**0.47**
PLT (K/µl)	**59**	**2633**	**990.7**	**1265.8**	**0.16**
PDW (fL)	**5.7**	**23.9**	**9.225**	**8.78**	**0.37**
MPV (fL)	**5.2**	**13.1**	**7.78**	**6.06**	**0.29**
WBC (K/µL)	**1.06**	**56.08**	**4.869333**	**2.654**	**0.09**
NEUT (K/µL)	**0.03**	**32.03**	**1.201333**	**0.956**	**0.60**
LYMPH (K/µL)	**0.12**	**23.46**	**3.126667**	**1.408**	**0.11**
MONO (K/µL)	**0**	**5.08**	**0.392667**	**0.222**	**0.56**
EO (K/µL)	**0**	**2.03**	**0.136**	**0.056**	**0.22**
BASO (K/µL)	**0**	**2.33**	**0.012**	**0.012**	**1**

Table I: Haematological characterisation of 3KO mice.

RBC = Total number of erythrocytes; HGB = Haemoglobin concentration; HCT = Haematocrit value: erythrocyte ratio of total blood volume; MCV = Mean erythrocyte volume in total sample; MCH = Mean haemoglobin volume per red blood cell (RBC); MCHC = Mean haemoglobin concentration of erythrocytes; PLT = platelet count per µl; PDW = Platelet distribution width; the degree of variation in size of the platelet population; MPV = Mean platelet volume; WBC = count of leukocytes per µl; NEUT = Neutrophil count per µl; LYMPH = Lymphocyte count per µl; MONO = Monocyte count per µl; EO = Eosinophil count per µl; BASO = Basophil count per µl.

Major Resources Table

In order to allow validation and replication of experiments, all essential research materials listed in the Methods should be included in the Major Resources Table below. Authors are encouraged to use public repositories for protocols, data, code, and other materials and provide persistent identifiers and/or links to repositories when available. Authors may add or delete rows as needed.

Animals (in vivo studies)

Species	Vendor or Source	Background Strain	Sex	Persistent ID / URL
C57BL6/J	The Jackson Laboratory		M/F	https://www.jax.org/strain/000664
BLAB/c	The Jackson Laboratory		M/F	https://www.jax.org/strain/000651

Genetically Modified Animals

	Species	Vendor or Source	Background Strain	Other Information	Persistent ID / URL
**Parent - Male**	3KO		C57BL6/J	Rezende et al., Antioxid Redox Signal. 2016; 24: 392-9.	https://pubmed.ncbi.nlm.nih.gov/25906178/
**Parent - Female**	3KO		C57BL6/J	Rezende et al., Antioxid Redox Signal. 2016; 24: 392-9.	https://pubmed.ncbi.nlm.nih.gov/25906178/
**Parent - Male**	NOX4-/-		C57BL6/J	Zhang et al., Proc Natl Acad Sci U S A 2010;107: 18121-6	https://pubmed.ncbi.nlm.nih.gov/20921387/
**Parent - Female**	NOX4-/-		C57BL6/J	Zhang et al., Proc Natl Acad Sci U S A 2010;107: 18121-6	https://pubmed.ncbi.nlm.nih.gov/20921387/

Antibodies

Target antigen	Vendor or Source	Catalog #	Working concentration	Lot # (preferred but not required)	Persistent ID / URL
Integrin αIIbβ3	Pharmingen	#553847			
GPVI	Thermo Fisher Scientifics	PA5-20583	1 µg/mL		
PE-conjugated anti-integrin αIIbβ3 (JON/A)	EMFRET	#M023-2	1/500 (no absolute concentration available)		
PE-conjugated anti- CD62P (Wug.E9)	EMFRET	#M130-2	1/200 (no absolute concentration available)		

DOI [to be added]

Phosphotyrosine antibody (4G10)	Millipore	# 05-321	1μg/ml		
Protein kinase C (PKC) phospho-substrates	Cell Signaling Technology	#2261,	1/1000 (no absolute concentration available)		
Extracellular Receptor Kinase (ERK)	Santa Cruz Biotechnology	sc-94	2μg/ml		
VASP (Ser239P)	Cell Signalling Technologies	#3114	1/1000 (no absolute concentration available)		
VASP	Cell Signalling Technologies	#3132	1/1000 (no absolute concentration available)		
Actin	Merck Millipore	#A5441	1/1000 (no absolute concentration available)		
GPIb (CD42b)	EMFRET	R300	1μg/g body weight		

DOI [to be added]
